# Exosomes maintain cellular homeostasis by excreting harmful DNA from cells

**DOI:** 10.1038/ncomms15287

**Published:** 2017-05-16

**Authors:** Akiko Takahashi, Ryo Okada, Koji Nagao, Yuka Kawamata, Aki Hanyu, Shin Yoshimoto, Masaki Takasugi, Sugiko Watanabe, Masato T Kanemaki, Chikashi Obuse, Eiji Hara

**Affiliations:** 1 The Cancer Institute, Japanese Foundation for Cancer Research (JFCR), Koto-ku, Tokyo 135-8550, Japan; 2 Graduate School of Life Science, Hokkaido University, Sapporo, Hokkaido 001-0021, Japan; 3 LSI Medience Corporation, Chiyoda-ku, Tokyo 101-8517, Japan; 4 Department of Molecular Microbiology, Research Institute for Microbial Diseases (RIMD), Osaka University, Suita, Osaka 565-0871, Japan; 5 Division of Molecular Cell Engineering, Department of Genetics, National Institute of Genetics, ROIS, SOKENDAI, Mishima, Shizuoka 411-8540, Japan; 6 PRESTO, Japan Science and Technology Agency (JST), Kawaguchi, Saitama 332-0012, Japan; 7 CREST, Japan Agency for Medical Research and Development (AMED), Chiyoda-ku, Tokyo 100-0004, Japan

## Abstract

Emerging evidence is revealing that exosomes contribute to many aspects of physiology and disease through intercellular communication. However, the biological roles of exosome secretion in exosome-secreting cells have remained largely unexplored. Here we show that exosome secretion plays a crucial role in maintaining cellular homeostasis in exosome-secreting cells. The inhibition of exosome secretion results in the accumulation of nuclear DNA in the cytoplasm, thereby causing the activation of cytoplasmic DNA sensing machinery. This event provokes the innate immune response, leading to reactive oxygen species (ROS)-dependent DNA damage response and thus induce senescence-like cell-cycle arrest or apoptosis in normal human cells. These results, in conjunction with observations that exosomes contain various lengths of chromosomal DNA fragments, indicate that exosome secretion maintains cellular homeostasis by removing harmful cytoplasmic DNA from cells. Together, these findings enhance our understanding of exosome biology, and provide valuable new insights into the control of cellular homeostasis.

## Abstract (web summary)

The role of exosomes in intercellular communication is well established, however less in known about the biological roles of exosome secretion in exosome-secreting cells. Here the authors show that exosome secretion controls cellular homeostasis in exosome-secreting cells by removing harmful cytoplasmic DNA from cells.

Higher eukaryotic cells are equipped with various potent self-defence mechanisms to preserve cellular homeostasis. One such mechanism is cellular senescence, which blocks the aberrant proliferation of cells at risk for neoplastic transformation, and is therefore believed to act as an important tumour suppressive mechanism^1^,^2^,^3^. Although irreversible cell-cycle arrest is traditionally considered as the major function of senescent cells^4^,^5^,^6^, recent studies have revealed some additional functions of senescent cells^1^,^2^,^3^. Most noteworthy, however, is the increased secretion of various secretory proteins, such as inflammatory cytokines, chemokines, growth factors and matrix metalloproteinases, into the surrounding extracellular fluid^7^,^8^,^9^,^10^. These newly recognised senescent phenotypes, termed the senescence-associated secretory phenotypes^9^, reportedly contribute to tumour suppression^7^,^8^, wound healing^11^, embryonic development^12^,^13^ and even tumorigenesis promotion^9^,^14^. Thus, senescence-associated secretory phenotypes appear to be beneficial or deleterious, depending on the biological context^15^,^16^.

In addition to secretory proteins, senescent cells also increase the secretion of a class of extracellular vesicles called ‘exosomes'^17^. Exosomes are endosomal membrane vesicles with diameters of ∼40–150 nm^18^,^19^,^20^. They originate in the late endosomal compartment from the inward budding of endosomal membranes, which generates intracellular multi-vesicular endosomes (MVEs)^18^,^21^. Pools of exosomes are packed in the MVEs and released into the extracellular space after the fusion of MVEs with the plasma membrane^18^,^21^,^22^. Emerging evidence has indicated that exosomes play important roles in intercellular communication, by serving as vehicles for transferring various cellular constituents, such as proteins, lipids and nucleic acids, between cells^23^,^24^,^25^,^26^,^27^. However, very little is known about the biological roles of exosome secretion in exosome-secreting cells^22^. Early hypotheses favoured the notion that exosomes may function as cellular garbage bags that expel unusable cellular constituents from cells^18^,^19^. However, this has not been explicitly proven^22^.

Since exosome secretion is reportedly increased in some senescent cells^17^, we examined the effects of the inhibition of exosome secretion in senescent cells. Surprisingly, we discovered that reducing exosome secretion provokes a reactive oxygen species (ROS)-dependent DNA damage response (DDR), in both senescent and non-senescent cells. Interestingly, the activation of ROS–DDR is a consequence of the accumulation of nuclear DNA fragments in the cytoplasm, where they are recognised by STING^28^,^29^,^30^,^31^, a cytoplasmic DNA sensor. This response was alleviated by the overexpression of a cytoplasmic DNase, the inhibition of STING activity or the inhibition of ROS generated by the interferon (IFN) pathway. These results, together with the observations that exosomes contain chromosomal DNA fragments, indicated that exosome secretion plays an important role in maintaining cellular homeostasis by removing harmful cytoplasmic DNA from cells, at least in certain types of normal human cells. Notably, the inhibition of exosome secretion in mouse liver, using hydrodynamics-based RNA interference (RNAi), revealed that this pathway also functions in this tissue, suggesting that this machinery may contribute more broadly to tissue homeostasis *in vivo.* Finally, we extended these findings to the antiviral activity of exosome secretion, which expels infected adenoviral DNA from cells. Thus, although we cannot exclude the possibilities that exosome secretion maintains cellular homeostasis by expelling not only cytoplasmic DNA but also other harmful cellular constituents from cells, our findings delineate a novel mechanism that links exosome secretion and cellular homeostasis.

## Results

### Exosome secretion maintains cellular homeostasis

**Figure 1:**
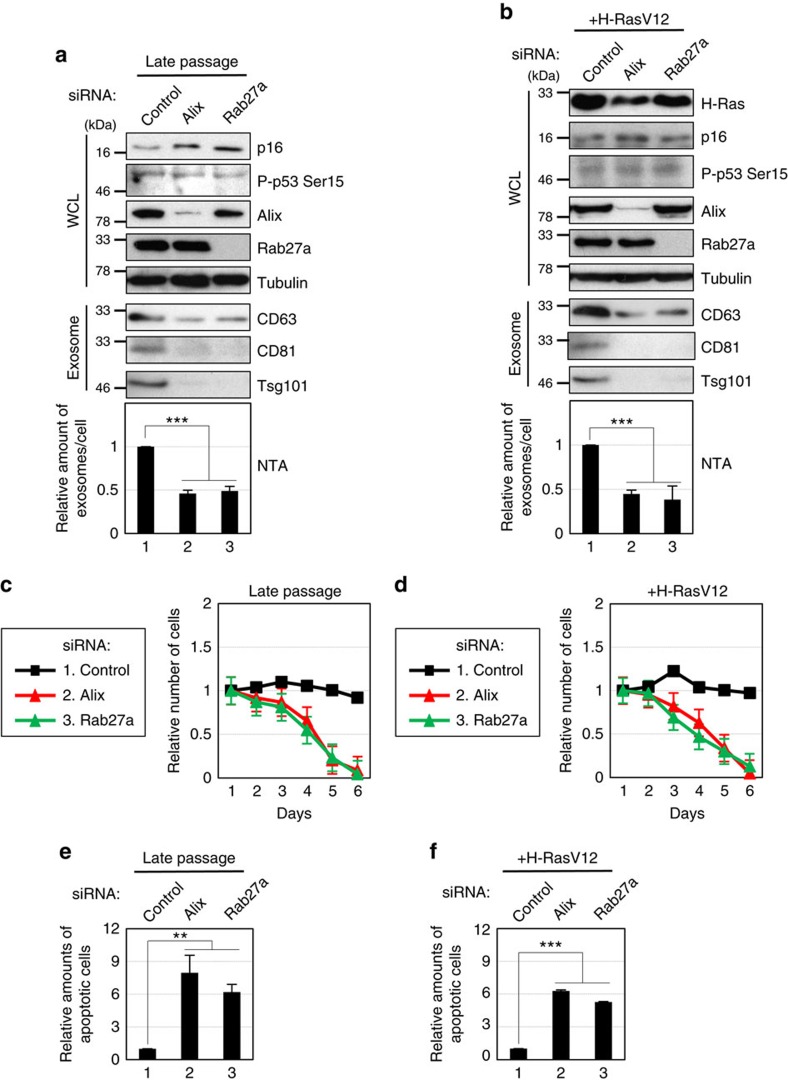
Inhibition of exosome secretion in senescent HDFs. (**a**,**b**) Senescent TIG-3 cells induced by serial passage (**a**) or oncogenic Ras expression (**b**) were transfected with validated siRNA oligos indicated at the top of the panel for twice at 2 day intervals. These cells were then subjected to western blotting using antibodies shown right (WCL) or to exosome isolation followed by western blotting using antibodies against canonical exosome markers shown right (exosome) and NanoSight analysis (NTA) for quantitative measurement of isolated exosome particles. The representative data from three independent experiments are shown. Tubulin was used as a loading control. (**c**–**f**) Senescent TIG-3 cells described in **a**,**b** were subjected to cell proliferation analysis (**c**,**d**) or to apoptosis analysis at day 4 (**e**,**f**). The representative data from three independent experiments are shown. For all graphs, error bars indicate mean±s.d. of triplicate measurements. (**P<0.01, ***P<0.001; one-way analysis of variance).

**Figure 2:**
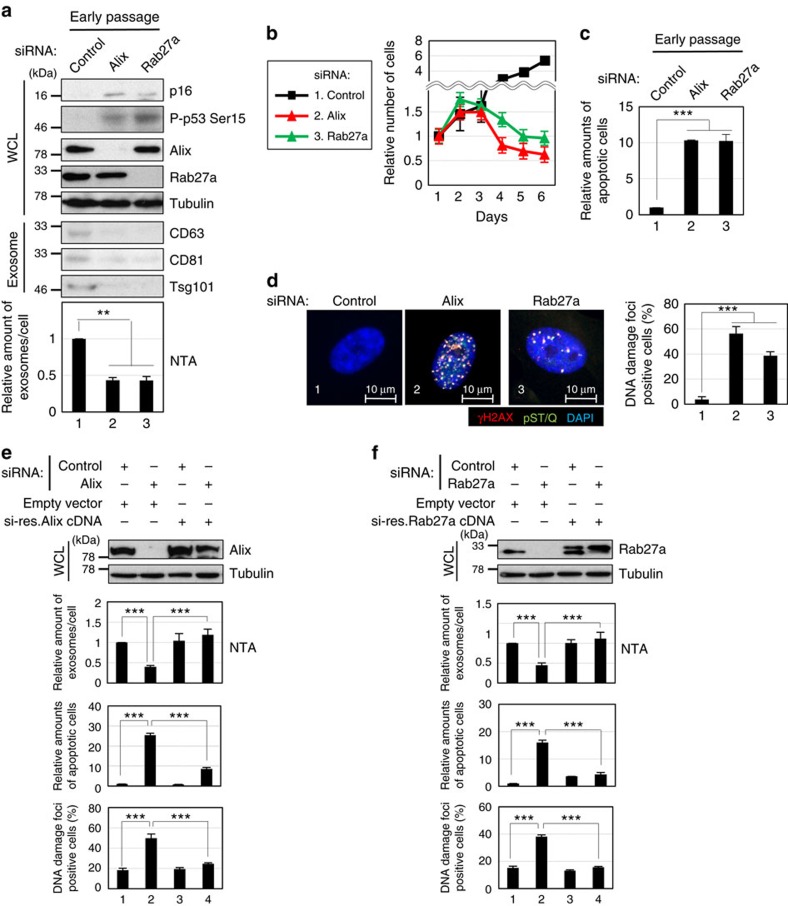
Inhibition of exosome secretion in pre-senescent HDFs. (**a**) Pre-senescent TIG-3 cells were subjected to transfection with indicated siRNA oligos twice (at 2 day intervals). These cells were then subjected to western blotting using antibodies shown right (WCL) or to exosome isolation followed by western blotting using antibodies against canonical exosome markers shown right (exosome) and NanoSight analysis (NTA) for quantitative measurement of isolated exosome particles. The representative data from three independent experiments are shown. Tubulin was used as a loading control. (**b**–**d**) Pre-senescent TIG-3 cells cultured under the conditions described in **a** were subjected to cell proliferation analysis (**b**), apoptosis analysis at day 4 (**c**) or to immunofluorescence staining for markers of DNA damage (γ-H2AX [red], phosphor-Ser/Thr ATM/ATR (pST/Q) substrate [green] and 4′,6-diamidino-2-phenylindole [blue]) (**d**). The representative data from three independent experiments are shown. The histograms indicate the percentage of nuclei that contain more than 3 foci positive for both γ-H2AX and pST/Q staining (**d**). At least 100 cells were scored per group (**d**). (**e**,**f**) Pre-senescent TIG-3 cells were infected with retrovirus encoding flag-tagged wild-type Alix or Rab27a protein containing a mutated siRNA cleavage site (lanes 3 and 4) or empty vector (lanes 1 and 2). After selection with puromycin, cells were transfected with indicated siRNA oligos and then subjected to western blotting using antibodies shown right, NanoSight analysis for quantitative measurement of isolated exosome particles, apoptosis analysis at day 4 or to immunofluorescence staining for markers of DNA damage. Tubulin was used as a loading control. The representative data from three independent experiments are shown. For all graphs, error bars indicate mean±s.d. of triplicate measurements. (**P<0.01. ***P<0.001; one-way analysis of variance).

To enhance our understanding of exosome biology, we first examined the effects of the inhibition of exosome secretion in senescent cells. Pre-senescent (early passage) normal human diploid fibroblasts (HDFs) were rendered senescent by either serial passage or ectopic expression of oncogenic Ras, the most established ways to induce cellular senescence^1^,^2^,^3^ (Supplementary Fig. 1a–c), and then exosomes were isolated by ultracentrifugation^32^. The isolated extracellular vesicles were confirmed to be exosomes, based on a nanoparticle tracking analysis (NTA), immuno-gold labelling for CD63, a well known exosome-associated protein, followed by transmission electron microscopy, and a western blotting analysis of canonical exosomal markers^33^ (Supplementary Fig. 1d–f). Consistent with a previous report^17^, exosome secretion was significantly increased in senescent cells, regardless of how the cellular senescence was induced (Supplementary Fig. 1f). We thus tried to inhibit exosome secretion by knocking down Alix or Rab27a, which are essential components of exosome biogenesis^34^ and secretion^35^, respectively, using previously validated small interfering RNAs (siRNAs)^36^,^37^ in senescent cells. In agreement with studies using several human cancer cell lines^34^,^35^,^36^,^37^,^38^, the depletion of either Alix or Rab27a substantially reduced exosome secretion, as judged by NTA and western blotting analyses of canonical exosomal markers (Fig. 1a,b). Interestingly, however, this was accompanied by apoptotic cell death (Fig. 1c–f), showing that there is an inverse correlation between the levels of exosome secretion and the incidence of apoptosis. To our surprise, moreover, a similar but less pronounced effect was also observed in pre-senescent cells (Fig. 2a–c). These results are unlikely to be the off-target effects of the siRNA oligos, since the introduction of siRNA-resistant Alix or Rab27a cDNA into the knockdown cells attenuated the effects of the siRNA oligos (Fig. 2e,f). Furthermore, two structurally unrelated chemical inhibitors of N-sphingomyelinase (nSMase), GW4869 and Spiroepoxide, which are well known inhibitors of exosome production^39^,^40^, also had the same effects in HDFs (Supplementary Fig. 2a–c) and other types of normal human cells (Supplementary Fig. 3a–c, e–g). Collectively, these results strongly suggested that exosome secretion plays a crucial role in maintaining cellular homeostasis at least in certain types of normal human cells, regardless of whether the cells are senescent.

### Exosome secretion prevents the aberrant activation of DDR

**Figure 3:**
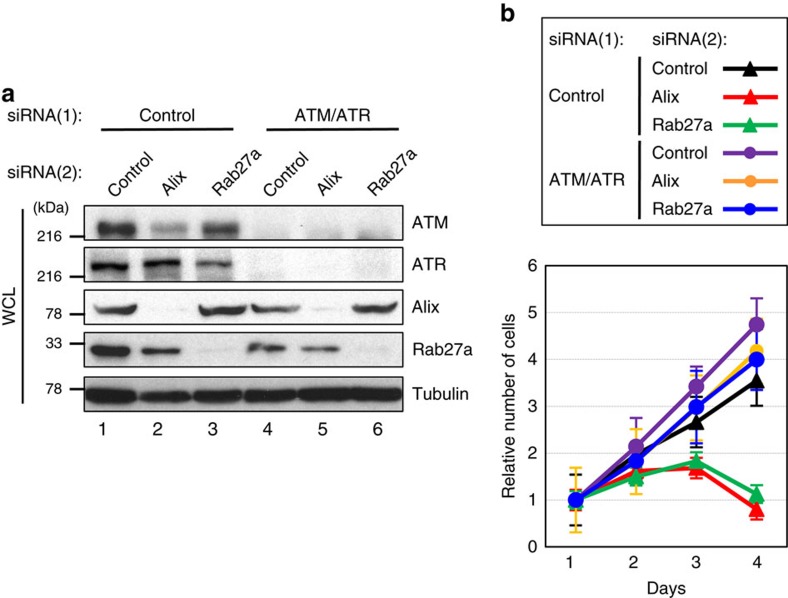
Exosomes secretion prevents ATM/ATR-dependent DDR. Pre-senescent TIG-3 cells were transfected with two different sets of validated siRNA oligos indicated at the top of the panel for twice at 2 day intervals. These cells were then subjected to western blotting using antibodies shown right (**a**) or to cell proliferation analysis (**b**). Tubulin was used as a loading control (**a**). The representative data from three independent experiments are shown. Error bars indicate mean±s.d. of triplicate measurements.

To substantiate this idea, we next sought to determine the underlying mechanisms by focusing on pre-senescent cells. Interestingly, we noted that the inhibition of exosome secretion provoked not only apoptosis but also senescence-like irreversible cell-cycle arrest in pre-senescent cells (Fig. 2a,b and Supplementary Figs 2b and 3b,f). Since the accumulation of DNA damage is known to cause apoptosis or cellular senescence, depending on the degree of DNA damage^1^,^2^,^3^,^41^, we tested whether the inhibition of exosome secretion provokes DNA damage in pre-senescent cells. Indeed, the reduction of exosome secretion by siRNAs or chemical inhibitors increased the signs of the DDR in normal human cells, as judged by γH2AX foci and the phosphorylation of the consensus target sequences (S/TQ) of Ataxia telangiectasia mutated (ATM) and Ataxia telangiectasia and Rad3 related protein (ATR), key components of the DDR pathway^42^ (Fig. 2d, Supplementary Figs 2d and 3d,h). Importantly, moreover, the simultaneous knockdowns of ATM and ATR using validated siRNA oligos^42^ abolished the onset of senescence-like cell-cycle arrest and apoptotic cell death in cells with Alix or Rab27a depletion (Fig. 3a,b). These results are also consistent with the observation that the inhibition of exosome secretion failed to induce senescence-like cell-cycle arrest and apoptotic cell death in human cancer cell lines, in which the DDR and/or cell-cycle checkpoint pathways are disrupted^31^ (Supplementary Fig. 4). Taken together, while additional mechanisms may participate, these data strongly suggested that exosome secretion plays a key role in the maintenance of cellular homeostasis by preventing the aberrant activation of DDR pathways, at least in certain types of normal human cells.

### Exosomes excrete harmful cytoplasmic DNA from cells

**Figure 4:**
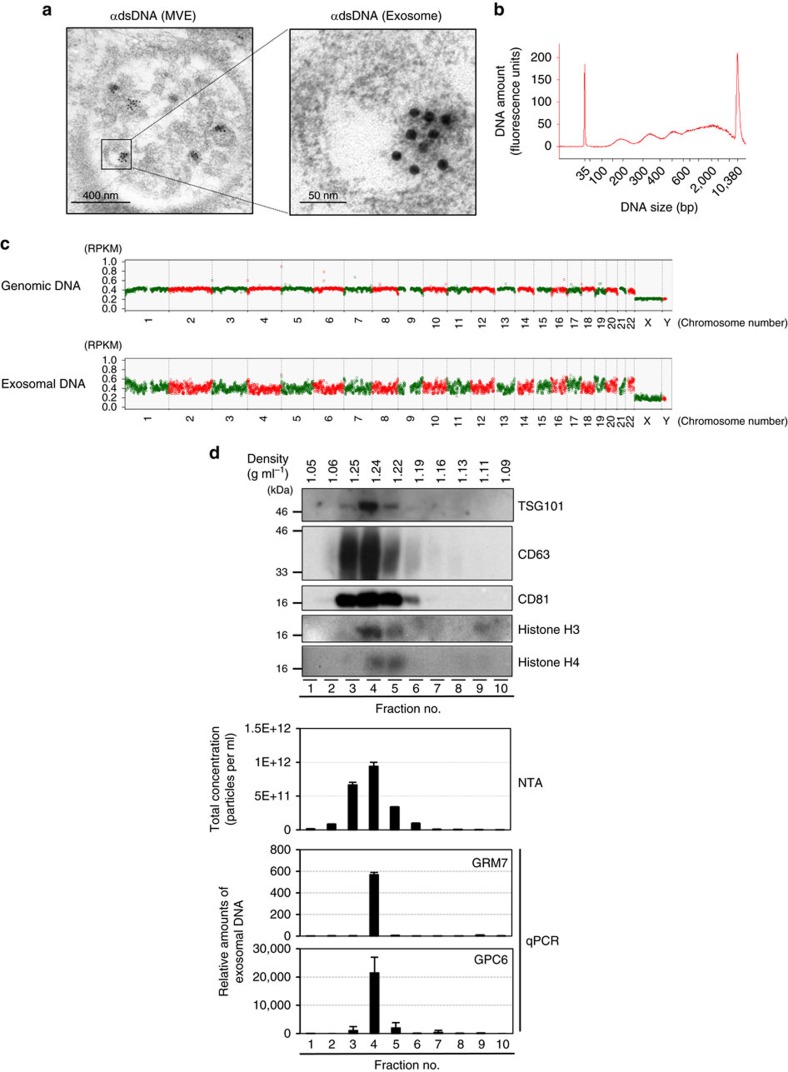
Exosomes contain chromosome fragments. (**a**) transmission electron microscopy micrograph of MVE in pre-senescent TIG-3 cells following immuno-gold labelling for dsDNA. Gold particles are depicted as black dots. Right image shows a digitally zoomed area of exosome. (**b**,**c**) Exosomal DNA isolated from pre-senescent TIG-3 cells were subjected to size distribution analysis using Electrophoresis Bioanalyzer system (**b**) or to deep sequencing analysis (**c**). Genomic DNA of TIG-3 cells are also subjected to deep sequencing analysis, as control (**c**). The read count of each 500-kb bin was normalized to RPKM and corrected by the mappability (**c**). (**d**) Purified exosomes from pre-senescent TIG-3 cells were subjected to sucrose density-gradient separation followed by western blotting using antibodies shown right, NanoSight analysis (NTA) for quantitative measurement of isolated exosome particles and quantitative PCR analysis for detection of genomic DNA fragments (GRM7 and GPC6).

To further explore this notion, we analysed how exosome secretion prevents the aberrant activation of DDR pathways. In seeking an explanation, we noted that exosomes released from HDFs have the potential to activate the DDR pathway in recipient pre-senescent HDFs, depending on the amount of added exosome (Supplementary Fig. 5). This result led us to propose that exosome secretion may prevent the aberrant activation of the DDR pathway, by excreting harmful cellular constituents from cells. Exosomes are known to contain various cellular components, such as proteins, lipids, RNA and DNA^21^,^22^,^23^,^24^,^43^. Among them, DNA is particularly interesting, because fragmented DNA is known to activate the DDR in normal cells^30^. Indeed, immuno-gold labelling of double-stranded DNA (dsDNA) followed by transmission electron microscopy revealed the presence of DNA in a certain proportion of the exosomes in MVEs (Fig. 4a). Moreover, electrophoresis and DNA sequencing analyses indicated that various lengths of dsDNA fragments spanning all chromosomes, but not from mitochondria, are present in the exosomes released from HDFs (Fig. 4b,c). These results are consistent with recent reports showing that exosomes secreted from human cancer cell lines contain dsDNA from all chromosomes^44^,^45^. Moreover, sucrose gradient separation of exosomes^32^ prepared from pre-senescent HDFs revealed that chromosomal DNA fragments were indeed present in the same fraction containing canonical exosome markers (Fig. 4d), further indicating that the exosomes secreted from HDFs contain chromosomal DNA fragments.

**Figure 5:**
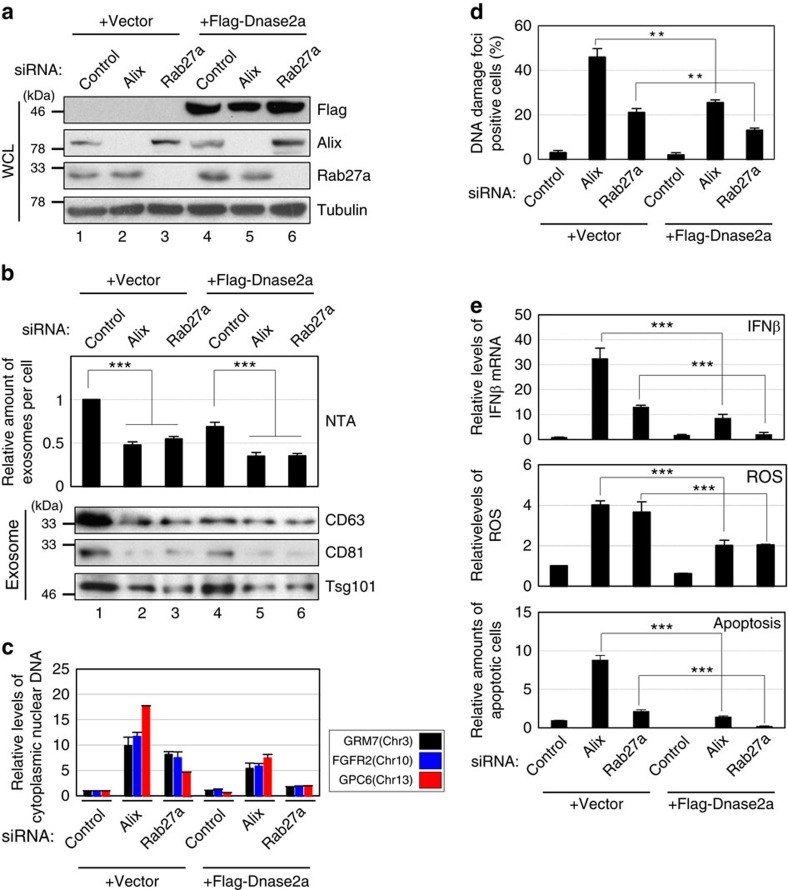
Overexpression of Dnase2a attenuated the effects of Alix or Rab27a knockdown in HDFs. Pre-senescent TIG-3 cells were infected with retrovirus encoding flag-tagged Dnase2a (lanes 4–6) or empty vector (lanes 1–3). After selection with puromycin, cells were transfected with indicated siRNA oligos and then subjected to western blotting using antibodies shown right (**a**), NanoSight analysis (NTA) for quantitative measurement of isolated exosome particles and western blotting using antibodies against canonical exosome markers shown right (exosome) (**b**), isolation of cytoplasmic fraction followed by quantitative PCR (qPCR) analysis of chromosomal DNA (**c**), immunofluorescence staining for markers of DNA damage (γ-H2AX [red], pST/Q (green) and 4′,6-diamidino-2-phenylindole (blue)) (**d**), qPCR analysis of IFNβ gene expression (**e**), analysis of intracellular ROS levels (**e**) or to apoptosis analysis at day 4 (**e**). Tubulin was used as a loading control (**a**). The histograms indicate the percentage of nuclei that contain more than 3 foci positive for both γ-H2AX and pST/Q staining (**d**). At least 100 cells were scored per group (**d**). The representative data from three independent experiments are shown. For all graphs, error bars indicate mean±s.d. of triplicate measurements. (**P<0.01. ***P<0.001; one-way analysis of variance).

Note that although exosomes are formed in the cytoplasm, damaged nuclear DNA is known to move into the cytoplasm^30^,^46^,^47^. Moreover, the amount of DNA in the exosomes (exosomal DNA) was substantially increased when pre-senescent HDFs were treated with DNA-damaging agents or rendered senescent, accompanied by the accumulation of nuclear DNA in the cytoplasm (Supplementary Fig. 6). It is therefore most likely that damaged nuclear DNA is the source of the exosomal DNA. Notably, the inhibition of exosome secretion also induced the cytoplasmic accumulation of nuclear DNA in pre-senescent HDFs (Supplementary Fig. 7), indicating that exosome secretion prevents the cytoplasmic accumulation of nuclear DNA. Consistent with this idea, the overexpression of Dnase2a, a lysosomal DNA endonuclease that targets dsDNA in the cytoplasm^46^,^48^, diminished the cytoplasmic accumulation of nuclear DNA and alleviated the activation of the DDR pathway in cells with Alix or Rab27a depletion (Fig. 5a–d). These results led us to propose that exosome secretion prevents the aberrant activation of DDR pathways, by excreting harmful cytoplasmic DNA of nuclear origin from the cells.

### Exosome secretion prevents aberrant innate immune response

**Figure 6:**
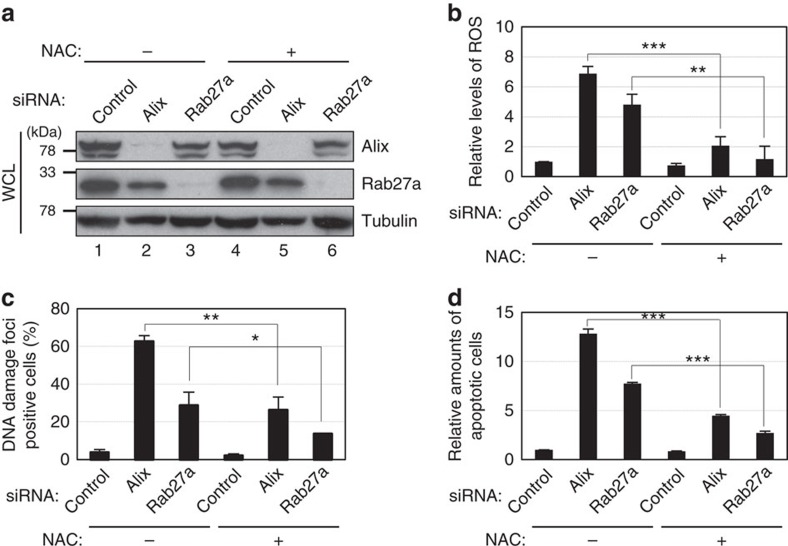
Reduction of ROS levels attenuated the effects of Alix or Rab27a knockdown in HDFs. Pre-senescent TIG-3 cells were transfected with validated siRNA oligos indicated at the top of the panel for two times at 2 day intervals in the presence or absence of 1 mM *N*-acetyl cysteine. These cells were then subjected to western blotting using antibodies shown right (**a**), analysis of intracellular ROS levels (**b**), immunofluorescence staining for markers of DNA damage (γ-H2AX (red), pST/Q (green) and 4′,6-diamidino-2-phenylindole (blue)) (**c**) or to apoptosis analysis (**d**). The histograms indicate the percentage of nuclei that contain more than 3 foci positive for both γ-H2AX and pST/Q staining (**c**). At least 100 cells were scored per group (**c**). The representative data from three independent experiments are shown. For all graphs, error bars indicate mean±s.d. of triplicate measurements. (*P<0.05. **P<0.01. ***P<0.001; one-way analysis of variance).

**Figure 7:**
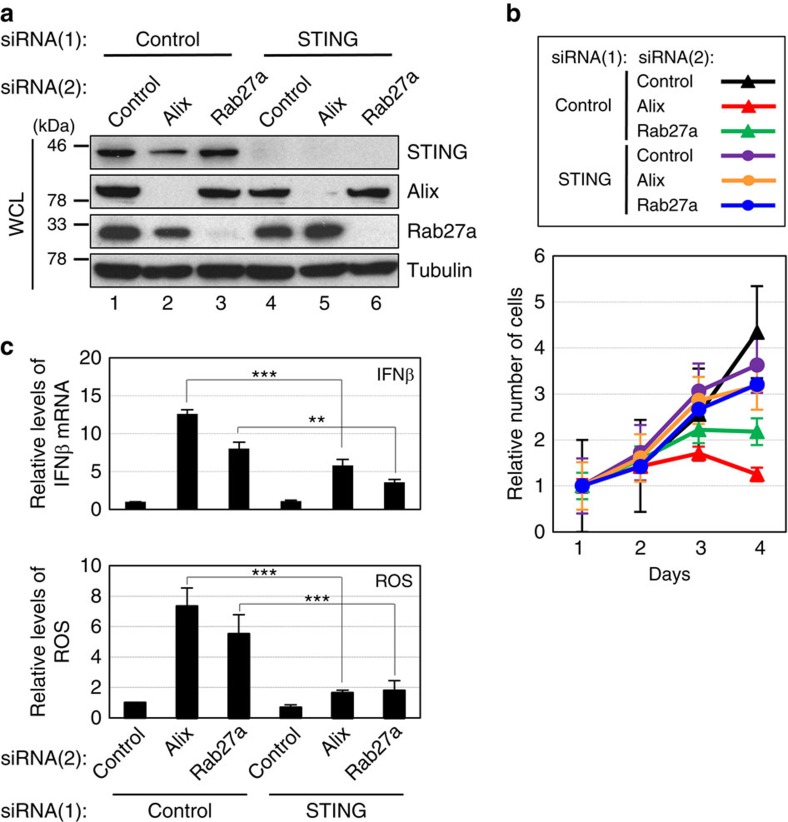
Depletion of STING attenuated the effects of Alix or Rab27a knockdown in HDFs. Pre-senescent TIG-3 cells were transfected with two different sets of validated siRNA oligos indicated at the top of the panel for three times at 2 day intervals. These cells were then subjected to western blotting using antibodies shown right (**a**), cell proliferation analysis (**b**) and quantitative PCR analysis of IFNβ gene expression or to analysis of intracellular ROS levels (**c**). Tubulin was used as a loading control (**a**). The representative data from three independent experiments are shown. For all graphs, error bars indicate mean±s.d. of triplicate measurements. (**P<0.01. ***P<0.001; one-way analysis of variance).

**Figure 8:**
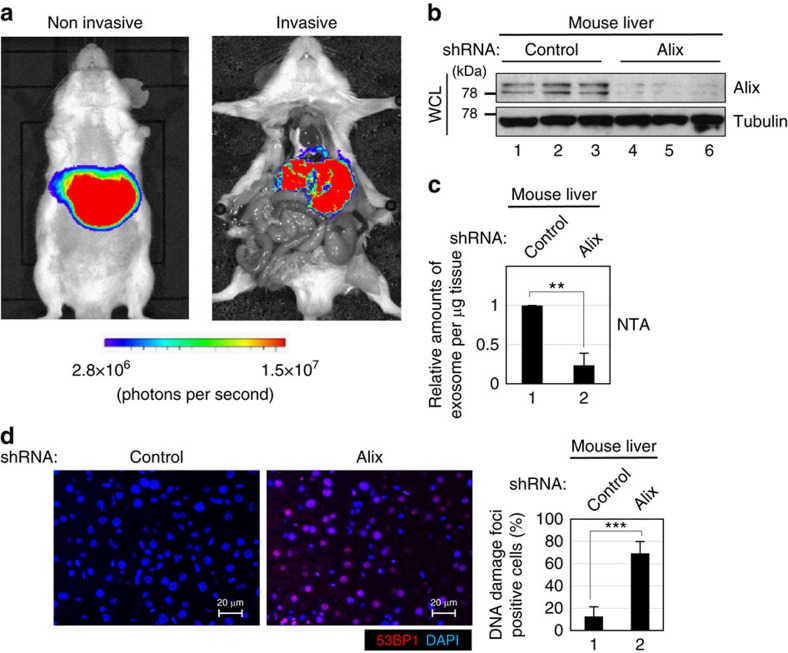
Inhibition of exosome secretion in mouse liver. ICR mice were subjected to hydrodynamic tail vein injection with plasmid encoding firefly luciferase or small hairpin RNA (shRNA) against Alix or control (*n*=3 per group). After 48 h, the mice transfected with firefly luciferase were subjected to i*n vivo* bioluminescent imaging for confirmation of the transfection efficiency (**a**), and then other mice were euthanized and livers were subjected to western blotting using antibodies shown right (**b**), NanoSight analysis (NTA) for quantitative measurement of isolated exosome particles (**c**) or to immunofluorescence analysis of liver section (**d**). Tubulin was used as a loading control (**b**). Section of livers were subjected to immunofluorescence staining for markers of DNA damage (53BP1 (red) and 4′,6-diamidino-2-phenylindole (blue)) (**d**). The histograms indicate the percentage of nuclei that contain more than 3 foci positive for 53BP1 staining. At least 100 cells were scored per group. The representative data from three independent experiments are shown. For all graphs, error bars indicate mean±s.d. of triplicate measurements. (**P<0.01. ***P<0.001; one-way analysis of variance).

These observations then raised the question of how the cytoplasmic accumulation of nuclear DNA provokes the DDR in normal human cells. Cytoplasmic DNA is reportedly detected by DNA sensors as a danger signal, resulting in the activation of the innate immune response, such as the type I IFN pathway^29^,^30^. Moreover, it has been reported that the IFN pathway can provoke the DDR by elevating the intracellular levels of ROS in HDFs^49^. These findings prompted us to examine if the inhibition of exosome secretion provokes the DDR, through the activation of the innate immune response by the cytoplasmic accumulation of nuclear DNA in normal human cells. Indeed, the IFN pathway was strikingly activated when exosome secretion was inhibited, coincident with the elevation of the intracellular levels of ROS in HDFs (Fig. 5e). Notably, these phenomena were attenuated when Dnase2a was overexpressed in HDFs (Fig. 5e), somewhat consistent with a previous observation that mice lacking *Dnase2* gene spontaneously produce high levels of type I IFNs and show embryonic lethality that is rescued by removing IFN receptor^50^. Moreover, the activation of DDR pathways and the onset of apoptosis were substantially diminished when ROS production was inhibited by *N*-acetyl-L-cysteine (NAC) treatment in HDFs with Alix or Rab27a depletion (Fig. 6). Similar results were also observed when STING, a cytoplasmic dsDNA sensor^29^,^30^,^31^, or its activator cGAS^31^,^51^ was knocked down, using previously validated siRNA oligos in HDFs with Alix or Rab27a depletion (Fig. 7 and Supplementary Fig. 8). Taken together, although other mechanisms may also be involved, the simplest explanation of our data is that exosome secretion preserves cellular homeostasis by blocking the aberrant activation of the innate immune response via preventing the cytoplasmic accumulation of harmful nuclear DNA, at least to some extent in normal human cells. It should also be noted that, as seen in murine fibroblasts (Supplementary Fig. 9), the depletion of Alix using hydrodynamics-based *in vivo* transfection provoked the reduction of exosome secretion and the aberrant activation of the DDR in mouse liver (Fig. 8), implying that this machinery may contribute more broadly to tissue homeostasis *in vivo.*

### Exosomes prevent the viral hijacking of cellular machinery

**Figure 9:**
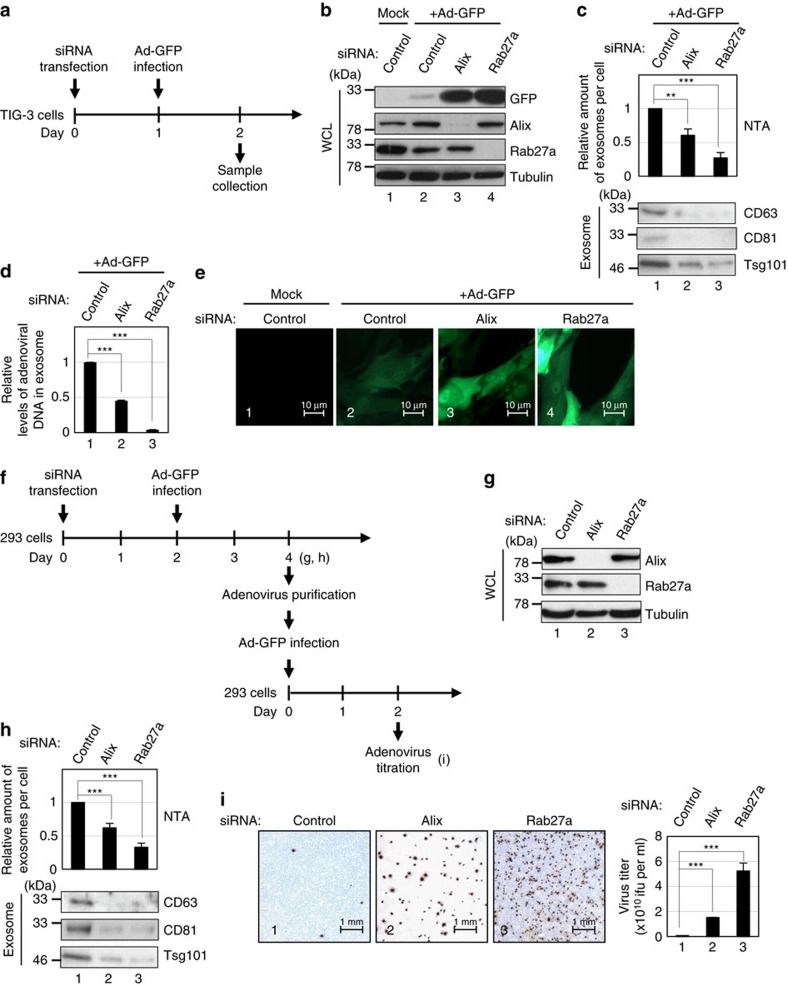
Exosome secretion prevents viral hijacking of cellular machinery. (**a**) Timeline of the experimental procedure. (**b**–**e**) Pre-senescent TIG-3 cells transfected with indicated siRNA oligos followed by infection with recombinant adenovirus encoding GFP (Ad-GFP) were subjected to western blotting using antibodies shown right (**b**), NanoSight analysis (NTA) and western blotting against canonical exosome markers for quantitative measurement of isolated exosome particles (**c**), quantitative measurement of isolated adenoviral DNA from exosome using quantitative PCR (**d**), or to microscopic analysis of GFP expression (**e**). The representative data from three independent experiments are shown. (**f**) Timeline of the experimental procedure. (**g**–**i**) 293 cells were transfected with indicated siRNA oligos followed by infection with Ad-GFP. These cells were then subjected to western blotting using antibodies shown right (**g**), NanoSight analysis (NTA) and western blotting against canonical exosome markers for quantitative measurement of isolated exosome particles (**h**) or to titration of generated Ad-GFP (**i**). The histograms indicate the virus titre (**i**). For all graphs, error bars indicate mean±s.d. of triplicate measurements. (**P<0.01. ***P<0.001; one-way analysis of variance).

**Figure 10:**
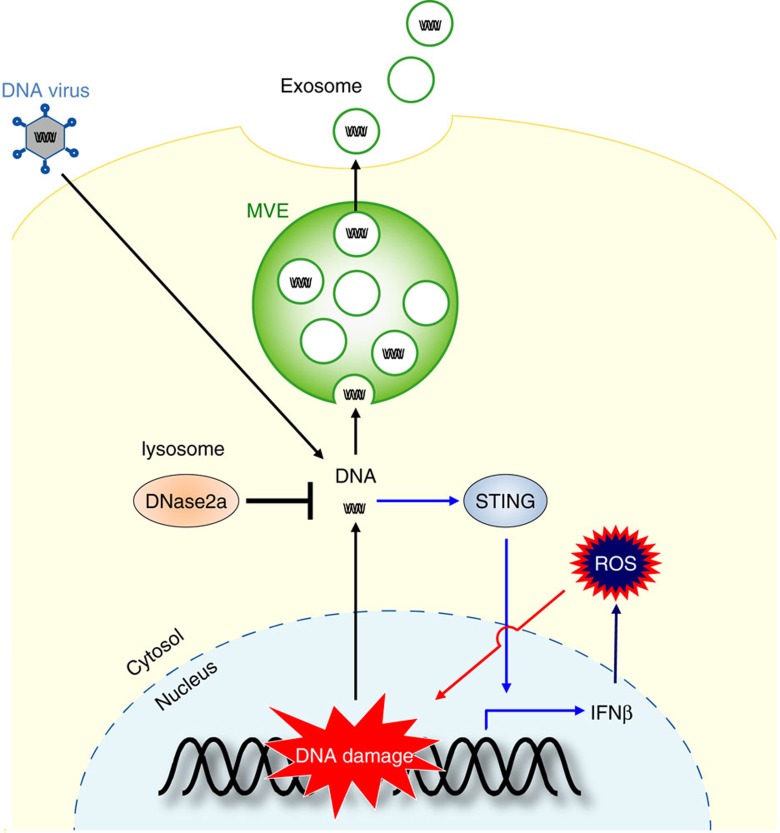
A model of exosome-mediated cellular homeostasis. The exosome secretion eliminates harmful cytoplasmic DNA from cells. The inhibition of exosome secretion causes the cytoplasmic accumulation of nuclear DNA, thereby causing the activation of STING, the cytoplasmic DNA sensing machinery. This event provokes the innate immune response, such as type I IFN pathway, leading to the elevation of the intracellular levels of ROS. In turn, this activates the DDR in normal human cells. This machinery may also play keys role in preventing viral hijacking of host cells by excreting viral DNA from cells.

Finally, to further clarify the biological significance of our findings, we analysed whether exosome secretion could also expel exogenous DNA, such as viral DNA, from cells. To this end, HDFs were infected with a recombinant adenovirus encoding green fluorescent protein (GFP), with or without the inhibition of exosome secretion (Fig. 9a–e). Indeed, the adenoviral DNA was excreted from the infected cells by exosomes (Fig. 9d). Interestingly, the levels of virally encoded protein expression were strikingly increased when exosome secretion was inhibited, as judged by the GFP expression levels (Fig. 9b,e), suggesting that exosome secretion also targets infected viral DNA for excretion from cells. We thus next tested if this machinery functions in preventing virus production in infected cells. Indeed, the inhibition of exosome secretion resulted in a dramatic increase in the production of infectious adenovirus in HEK293 cells (Fig. 9f–i), indicating that exosome secretion also plays an important role in preventing the viral hijacking of cellular machinery, although other mechanisms are also likely to be involved (see model in Fig. 10). These results are somewhat similar to those observed in latent Epstein–Barr virus-infected cells where sorting and secretion of pro-inflammatory viral RNA via exosomes prevent activation of IFN-β pathway^52^. Collectively, these results revealed an additional mechanism for the antiviral activity of exosomes^40^, further illustrating the biological significance of the exosome-mediated removal of harmful DNA from cells.

## Discussion

Exosome secretion had initially been proposed as a mechanism to maintain cellular homeostasis, by removing excess or obsolete molecules from cells^18^,^19^. However, emerging evidence has revealed that the secretion of exosomes also plays important roles in mediating cell-to-cell communication, by activating various signalling pathways in cells with which they fuse and interact^21^,^22^,^23^,^24^,^25^,^26^,^27^. Despite considerable progress in understanding how cell-to-cell communication is implemented by exosomes^22^,^23^,^24^,^25^,^26^,^27^,^36^,^37^, far less is known about how exosome secretion maintains cellular homeostasis in exosome-secreting cells. In this study, we provide evidence that the inhibition of exosome secretion, pharmacologically or by RNAi, activates the ATM/ATR-dependent DDR in both senescent and non-senescent normal human cells. This response is at least partly due to the accumulation of nuclear DNA fragments in the cytoplasm, since the reduction of cytoplasmic nuclear DNA by the overexpression of Dnase2a or the inhibition of the STING/cGAS cytoplasmic DNA sensor by RNAi substantially alleviated this response. Although other mechanisms may also be involved, the simplest explanation of our data is that exosome secretion preserves cellular homeostasis by blocking the aberrant activation of the DDR via preventing the cytoplasmic accumulation of harmful nuclear DNA, at least to some extent in normal cells (see model in Fig. 10). This mechanism appears to become more important in senescent cells, presumably because nuclear DNA tends to accumulate in the cytoplasm in senescent cells^47^ (see also Supplementary Fig. 6g).

However, neither Alix nor Rab27a nor nSMase functions exclusively in exosome secretion^34^,^35^,^39^. For example, Alix is known to play key roles in cytokinetic abscission^53^. Thus, it is possible that additional mechanisms may also be involved in the activation of the DDR pathway in our experimental setting. Nevertheless, we observed exactly the same effects when we blocked the functions of these proteins (Figs 1 and 2, Supplementary Figs 2 and 3) and other proteins (Tsg101 (ref. 17), Rab27b (ref. 35) or Slp4 (ref. 35)) known to be involved in exosome biogenesis or secretion in HDFs (Supplementary Fig. 10). Moreover, we did not see substantial increase in the frequency of multinucleate cells, a sign of cytokinetic failure, in HDFs with Alix depletion (Supplementary Fig. 11). Furthermore, the purified exosomes contained genomic DNA fragments (Fig. 4) and had the potential to provoke the DDR in recipient normal human cells, depending on the amounts of added exosomes (Supplementary Fig. 5). Thus, although we cannot yet completely rule out the possibility that additional mechanism(s) may also be involved, it is most likely that exosome secretion maintains cellular homeostasis by excreting harmful cytoplasmic DNA, at least to some extent, in normal cells. It is also worth noting that neither apoptosis nor necrosis was observed in control pre-senescent HDFs (Fig. 2c, lane 1), precluding the possibility that the genomic DNA fragments observed in our exosome fractions originated from apoptotic bodies. Along a similar line, the inhibition of apoptosis by Z-VAD, a pan caspase inhibitor, did not have any impact on the appearance of the DDR in pre-senescent HDFs treated with exosome inhibitors (Supplementary Fig. 12). Collectively, these results indicate that the DDR provoked by the blockage of exosome secretion is not simply a consequence of the uptake of apoptotic DNA fragments through the endocytosis of apoptotic bodies in HDFs.

It has been shown that the deficiency of Dnase2a leads to accumulation of damaged self DNA and induction of pro-inflammatory cytokine pathways in murine cells^46^. Moreover, removal of damaged self DNA by Dnase2a was shown to require autophagy-mediated delivery of the DNA to lysosomes^46^. These notions, in conjunction with a very recent observation that prevention of autophagy-lysosome fusion increases exosome secretion^54^, imply that exosome secretion and autophagy may act in a complementary manner to remove pro-inflammatory DNA from cells (see model in Fig. 10).

The obvious remaining questions are the origins of the exosomal DNAs and how are they generated. Notably, cells in G0 phase of the cell-cycle are more resistant to the inhibition of exosome secretion, as compared to those in the proliferating phase in pre-senescent HDFs (Supplementary Fig. 13). Thus, it is very likely that exosomal DNA fragments are generated by the conservative homologous recombination^55^ that occurs preferentially in the late S, G2 and M phases of the cell-cycle in pre-senescent cells. These observations also suggest that the cell-cycle status may determine whether the inhibition of exosome secretion in pre-senescent cells drives the cells into apoptosis or senescence-like cell-cycle arrest. However, because senescent cells are non-proliferative, exosomal DNA fragments are likely to be generated by different mechanism(s) in these cells. This idea is somewhat consistent with recent observations that damaged nuclear DNA tends to move into the cytoplasm^30^,^46^,^47^. It is also worth mentioning that at least a certain proportion of the exosomal DNA was bound to histones (Supplementary Fig. 14), and histones are reportedly abundant in exosomes^56^,^57^ (see also Fig. 4d). Thus, it is tempting to speculate that nuclear DNA may be loaded into exosomes through histones.

In summary, while the precise mechanisms underlying the nuclear DNA loading into exosomes require further clarification, our results support a model in which exosome secretion maintains cellular homeostasis by removing harmful cytoplasmic DNA from cells, at least to some extent, in certain types of normal cells (see model in Fig. 10). These results reveal an unexpected role of exosome secretion, and provide new insights into the maintenance of cellular homeostasis in normal cells, opening up new possibilities for its control.

## Methods

### Cell culture

TIG-3 cells^5^,^42^, HEK-293T cells, HeLa cells and U2OS cells were obtained from Japanese Cancer Research Resources Bank (JCRB) and mouse embryonic fibroblasts (MEFs) were established from day 13.5 mouse embryos. These cells were cultured in Dulbecco's Modified Eagle's medium supplemented with 10% foetal bovine serum. HRPE cells (Lonza Inc.) and HEK cells (Cell Applications Inc.) were cultured according to the manufacturer's instructions. Early passage TIG-3 cells (<40 population doublings) were used as growing cells, and late passage TIG-3 cells (>70 population doublings) that ceased proliferation were used as replicative senescent cells. For retroviral infection, TIG-3 cells were rendered sensitive to infection by ecotropic retroviruses^5^. Cells were then infected with recombinant retroviruses encoding Ras V12 (in pBabe–puro^58^), DNase2 (in pMarX–puro^59^), and siRNA-resistant Alix and Rab27a (in pMarX–puro). MEFs were infected with recombinant retroviruses encoding shRNA against *Alix* (in pRetrosuper–puro^60^). After puromycin selection, pools of drug-resistant cells were analysed 7 days after infection. In some experiments, cells were treated with an N-SMase inhibitor GW4869 (SIGMA), Spiroepoxide (Santa Cruz), doxorubicin (Wako), Z-VAD-FMK (Promega) or *N*-acetyl-L-cysteine (SIGMA). We have confirmed the absence of mycoplasma contamination in our tissue culture cells.

### Cell proliferation assay

Cells were plated on 35 mm dishes with 2 mm grids (Thermo Fisher Scientific). The number of cells in each grid was counted every day, and the relative number of cells was calculated based on an adjusted cell number at day 1 set at 1.0.

### Apoptosis assay

Apoptotic cells were washed with binding buffer and stained with an fluorescein isothiocyanate-Annexin V solution using an apoptotic/healthy cells detection kit (PromoKine). After an incubation at room temperature for 15 min, fluorescence was measured with a Wallac ARVO 1420 Multilabel counter (PerkinElmer Co., Ltd.).

### Exosome isolation from cells

For exosome isolation, cells were incubated in Dulbecco's Modified Eagle's medium with 5% foetal bovine serum, depleted of microvesicles, for 48 h. The cell supernatants were collected and centrifuged at 300 g for 5 min to eliminate cells, and then at 2,000*g* for 10 min to remove the cellular debris. The supernatant was then centrifuged at 10,000*g* for 30 min, followed by filtration through a 0.22-μm pore filter (Sigma) to remove the contaminating apoptotic bodies, shedded vesicles and cell debris. The collected supernatant was then ultracentrifuged at 100,000*g* for 70 min and the precipitate was rinsed with PBS twice, as previously described^23^,^32^. For sucrose density-gradient separation, purified exosome fractions were further subjected to sucrose density-gradient centrifugation^32^. In brief, 0.5 ml of purified exosome fractions were suspended in 1.5 ml of 3.3 M sucrose in 20 mM HEPES/NaOH at pH 7.2 and loaded in a SW 32 Ti tube (Beckman Coulter), and 30 ml of a continuous sucrose gradient, from 2.0 M to 0.25 M sucrose in 20 mM HEPES/NaOH at pH 7.2, was layered on top. Tubes were centrifuged at 100,000*g* for 18 h at 4 °C. Ten fractions with equal volumes (3.2 ml) were collected from the top of the gradient. The density for each fraction after ultracentrifugation was determined using a refractometer (RX-5000α, Atago Co. Ltd, Tokyo, Japan). All fractions were resuspended in 30 ml PBS and were again centrifuged at 100,000 g for 70 min at 4 °C. The washed pellet was resuspended in 0.5 ml PBS. The collected fractions were stored at 4 °C until further analysis. The size distribution and concentration of the exosomes were determined by NTA, using a NanoSight LM10 system (NanoSight Ltd.).

### Exosome isolation from mouse tissue

Fresh mouse liver sections (40 μg) were washed with 40 ml of PBS and then incubated with 1.5 ml of RPMI-1640 medium (Nacalai Tesque) including antibiotics (Sigma), at 37 °C for 4 h with agitation in CO_2_ incubator. The medium was collected and centrifuged at 2,000*g* for 15 min and again 12,000*g* for 15 min, followed by filtration through a 0.22-μm pore filter (Sigma). The supernatant was then subjected to ultracentrifugation at 100,000*g* for 70 min, and the precipitate was rinsed with PBS twice. The size distribution and concentration of the exosomes were determined using a NanoSight LM10 system (NanoSight Ltd.).

### Quantitative measurement of isolated exosomal DNA

To reduce external DNA contamination, prior to DNA extraction, exosomes were treated with DNase I (Roche Inc.) and Exonuclease III (Takara Inc.), according to the manufacturers' instructions^43^. After heat inactivation, the exosomal DNA was purified by Proteinase K (Wako) treatment. The amount of dsDNA was determined using an Agilent High Sensitivity DNA kit (Agilent Technologies) or a QuantiFluor dsDNA System (Promega).

### Cytoplasmic nuclear DNA analysis

Cytoplasmic fractions were obtained using an NE-PER Nuclear and Cytoplasmic Extraction Kit (Thermo Fisher Scientific). Cytoplasmic DNA was purified by Proteinase K (Wako) treatment. The amount of nuclear DNA was determined by quantitative real-time PCR, using three different sets of primers designed for different chromosomes (GRM7, FGFR2 and GPC6).

### Deep sequencing of exosomal DNA

The deep sequencing analysis was performed as previously described^61^. Briefly, 300 ng portions of exosomal DNA and genomic DNA were sheared using a Bioruptor USD-250 bath sonicator (Cosmo-Bio; 96 sonications of 15 s with 30-s intervals at 250 W). Libraries were prepared according to the manufacturer's instructions (Illumina). Fifteen nanogram of sheared DNA was end-repaired using a mix of Klenow DNA polymerase, T4 DNA polymerase and T4 polynucleotide kinase (NEB), tailed with an ‘A' base using Klenow Fragment 3′–5′ exo minus (NEB), and ligated with the Illumina single-end adaptor, using a DNA ligation Kit (TaKaRa). Adaptor-ligated fragments of ∼400 bp were purified using the E-gel SizeSelect system (Life Technologies), and were subjected to 15 cycles of PCR amplification using KOD FX polymerase (TOYOBO). To remove the remaining PCR primers, the amplified products were further purified using AMPure XP Kits (Beckman Coulter). Libraries were quantified using a BioAnalyzer 2100 with a High Sensitivity DNA Kit (Agilent) and sequenced using an Illumina Genome Analyzer IIx to generate 4 × 10^7^ 41-bp single-end reads. The sequence reads were aligned to the human reference genome, UCSC hg18 including a single unit of human ribosomal DNA repeats, using BWA with default parameters rbin. Reads with low mapping quality (<20) were discarded, to allow reads mapped to unique genomic positions to be considered with confidence. Possible PCR duplicates were removed using samtools. We divided each chromosome into non-overlapping 500-kb bins and calculated the number of mapped reads per kilo base per million reads (RPKM) for each bin. To correct a mappability bias, all possible 41-mer sequences of the reference genome were mapped back in the same manner as the sequenced reads and the RPKM value was divided by the fraction of mappable bases for each bin.

### Electron microscopy

Exosomes isolated from TIG-3 cells were absorbed to formvar carbon-coated nickel grids and immune-labelled with an anti-CD63 antibody (BD, 556019), followed by 5 nm of a gold-labelled secondary antibody (British BioCell International Ltd.). The samples were fixed in 2% glutaraldehyde in 0.1 M phosphate buffer, and a 2% phospho tungstic acid solution (pH 7.0) was used for negative staining. For the observation of cellular dsDNA, TIG-3 cells were plated on the gold disks and frozen in liquid propane at −175 °C. The samples were freeze substituted with 0.2% glutaraldehyde in acetone and 2% distilled water at −80 °C for 2 days. After dehydration, the samples were embedded into resin (LR White, London Resin Co. Ltd.) and ultra-thin sectioned at 80 nm using an ultramicrotome (Ultracut UCT, Leica). The samples were immunolabelled with an anti-dsDNA antibody (Santa Cruz, sc-58749) in normal goat serum and 1% BSA, followed by 10 nm gold-labelled secondary antibody. The grids were placed in 2% glutaraldehyde in 0.1 M phosphate buffer and dried. They were stained with 2% uranyl acetate for 15 min and a Lead stain solution (SIGMA). The samples were observed with a transmission electron microscope (JEM-1400Plus, JEOL Ltd.) at 80 kV. Digital images were obtained with a CCD camera (VELETA, Olympus Soft imaging solutions GmbH).

### Fluorescence microscopic analysis

Immunofluorescence analysis was performed using antibodies against γ-H2AX (1:1,000, Millipore, 05-636), phosphor-(Ser/Thr) ATM/ATR substrate (1:500, Cell Signaling Technology, 2851) and 53BP1 (1:1,000, Santa Cruz, sc-22760; 1:1,000, abcam, ab36823). DNA was stained with 2 mg ml^−1^ 4′,6-diamidino-2-phenylindole (Dojindo). Fluorescence images were observed and photographed using an immunofluorescence microscope (Carl Zeiss)^14^,^62^.

### RNAi

RNAi was performed by the transfection of siRNA oligos using the Lipofectamine RNAiMAX transfection reagent (Thermo Fisher Scientific), according to the manufacturer's instructions. The sequences of the siRNA oligos were as follows. Alix^34^,^63^: 5′-GAACCUGGAUAAUGAUGAA-3′. Rab27a (ref. 35): 5′-GCUGCCAAUGGGACAAACA-3′.Rab27b (ref. 35): 5′-CCCAAAUUCAUCACUACAGUA-3′. Tsg101 (ref. 64): 5′-CCUCCAGUCUUCUCUCGUC-3′. ATM^42^: 5′-UGAAGAUGGUGCUCAUAAA-3′. ATR^42^: 5′-CCUCCGUGAUGUUGCUUGA-3′. cGAS^51^: 5′-GGAAGGAAAUGGUUUCCAA-3′. MISSION siRNA targeting for Slp4/Sytl4 (5′-CUGUUAAUCCACUAUAUGA-3′) was purchased by Sigma Genosys. ON-TARGETplus siRNAs (Dharmacon) and non-targeting control siRNA were used to target Sting^65^,^66^,^67^ and Alix^34^. Knockdown efficiency was confirmed by quantitative real-time PCR. For knockdown of Alix or Rab27a in MEFs and mouse liver, the target sequences of the shRNA used were as follows: *mouse Alix*, 5′-GAACCUGGAUAAUGAUGAA-3′ (ref. 63); control, 5′-CAUUGCUAUAGAGGCAGAU-3′ (ref. 62).

### Plasmids

The epitope tagged cDNAs of DNase2, Alix and Rab27a were cloned into the pMarX–puro retrovirus vector^5^,^42^,^59^. To generate siRNA-resistant Alix and Rab27a mutants, seven- or six-point mutations, which do not change the encoded amino acids, were introduced into the 21-nucleotide target regions of the Alix and Rab27a cDNAs, using a Quick Change Site-directed Mutagenesis kit (Stratagene). For the construction of siRNA-resistant mutants, the primer sequences were as follows: *Alix,* 5′-GCAGCAGAACAAAATCTCGACAACGACGAGGGATTGAAAATCG-3′ (forward) and 5′-CGATTTTCAATCCCTCGTCGTTGTCGAGATTTTGTTCTGCTGC-3′ (reverse); and *Rab27a,* 5′-CTTTGAAACTAGTGCAGCGAACGGTACGAATATAAGCCAAGC-3′ (forward) and 5′-GCTTGGCTTATATTCGTACCGTTCGCTGCACTAGTTTCAAAG-3′ (reverse). All cDNAs were sequenced on a Genetic Analyzer 3130 (Applied Biosystems) using a BigDye Terminator v3.1 Cycle Sequencing Kit (Applied Biosystems).

### Quantitative real-time PCR

Total RNA was extracted from cultured cells using a mirVana kit (Thermo Fisher Scientific), and then subjected to reverse transcription using a PrimeScript RT reagent kit (TaKaRa). Quantitative real-time RT-PCR was performed on a StepOnePlus PCR system (Applied Biosystems) using SYBR Premix Ex Taq (TaKaRa). The PCR primer sequences were listed in Supplementary Table 1. The means±s.d. of three independent experiments are shown.

### Western blotting

For western blotting analysis, cells or mouse liver tissues were lysed in lysis buffer (50 mM Hepes, pH 7.5, 150 mM NaCl, 1 mM EDTA, 2.5 mM EGTA, 10% glycerol, 0.1% Tween20, 10 mM β-glycerophosphate) with 1% Protease inhibitor cocktail (Nacalai Tesque)^5^,^42^. The protein concentration was determined using a DC Protein Assay (Bio-Rad), and proteins were separated by SDS–polyacrylamide gel electrophoresis and transferred onto a polyvinylidene difluoride membrane (EMD Millipore). After blocking with 5% milk, membranes were probed with primary antibodies (1:1,000) as follows: anti-H-Ras (Santa Cruz, sc-29), p16 (IBL, 11104), G9a (Cell Signaling Technology, 3306), GLP (MBL, D220-3), DNMT1 (BD Transduction Laboratories, 612618), Lamin B (Santa Cruz, sc-6217), phospho-p53 (Ser15; Cell Signaling Technology, 9286), ATM (SIGMA, A1106), ATR (MABI, 123-3), Alix (Proteintech, 12422), Rab27a (Proteintech, 17817), Rab27b (Proteintech, 13412), Slp4 (Proteintech, 12128), STING (Cell Signaling Technology, 13647), cGAS (Cell Signaling Technology, 15102), Caspase3 (Cell Signaling Technology, 9662), CD63 (BD, 556019), CD81 (Cosmo-Bio, SHI-EXO-M03), Tsg101 (Proteintech, 14497), Histone H3 (abcam, ab1791), Histone H4 (abcam, 10158), DNase2 (ProSci, 2059), GFP (Clontech, 632460) and α-tubulin (SIGMA, T9026). The membranes were then incubated with secondary antibodies (GE Healthcare; 1:2,000) and visualised with the Chemi-Lumi One reagent (Nacalai Tesque). Full immunoblots are provided in Supplementary Fig. 15.

### ROS analysis

To assess the levels of intracellular ROS generation, cells were incubated with 20 μM DCF-DA (Calbiochem) at 37 °C for 20 min. The peak excitation wavelength for oxidised DCF was 488 nm and that for emission was 525 nm, measured by using a Wallac ARVO 1420 Multilabel counter (PerkinElmer Co., Ltd.)^5^.

### Adenovirus infection

TIG-3 cells were transfected with siRNA oligo and the next day cells were infected with recombinant adenovirus (Ad5) encoding GFP^68^, at a multiplicity of infection of 10. For viral titration analysis, 293 cells were transfected with siRNA oligo and 2 days later cells were infected with Ad5 encoding GFP, and the virus titre was determined using an Adeno-X Rapid Titer Kit (Clontech).

### Animal experiments

Hydrodynamics-based transfection was performed using 30-day-old male ICR mice^69^. In brief, 20 μg of plasmid encoding firefly luciferase or 20 μg of shRNA plasmid against *Alix* or control plasmid, in 2.5 ml saline, was injected into the tail vein of mice over a short duration of 5–7 s, to facilitate the uptake of plasmid DNA in the liver^69^. Forty-eight hours later, mice transfected with luciferase were subjected to *in vivo* bioluminescent imaging^62^,^70^ for confirmation of the transfection efficiency, and mice transfected with shRNA were euthanized and the liver sections were subjected to exosome collection, western blotting or immunofluorescence analysis. The sample size used in this study was determined based on the expense of data collection, and the requirement for sufficient statistical significance. Randomisation and blinding were not used in this study. Mice with body weights between 24.2 and 26.2 g at the age of 30 days were used for experiments. All animal care was performed according to the protocols approved by the Committee for the Use and Care of Experimental Animals of the Japanese Foundation for Cancer Research.

### Statistical analysis

Statistical significance was determined using a Student's *t*-test and one-way analysis of variance. *P* values <0.05 were considered significant.

### Data availability

Sequencing data of exosomal DNA has been deposited in the DDBJ sequence read archive under accession number DRA005580. The authors declare that all other data are available from the authors upon request.

## Additional information

**How to cite this article:** Takahashi, A. *et al*. Exosomes maintain cellular homeostasis by excreting harmful DNA from cells. *Nat. Commun.* **8**, 15287 doi: 10.1038/ncomms15287 (2017).

**Publisher's note:** Springer Nature remains neutral with regard to jurisdictional claims in published maps and institutional affiliations.

## Supplementary Material

Supplementary Information Supplementary Figures, Supplementary Table and Supplementary References
