# Publisher Correction: Exosomes maintain cellular homeostasis by excreting harmful DNA from cells

**DOI:** 10.1038/s41467-018-06613-3

**Published:** 2018-10-08

**Authors:** Akiko Takahashi, Ryo Okada, Koji Nagao, Yuka Kawamata, Aki Hanyu, Shin Yoshimoto, Masaki Takasugi, Sugiko Watanabe, Masato T. Kanemaki, Chikashi Obuse, Eiji Hara

**Affiliations:** 1 The Cancer Institute, Japanese Foundation for Cancer Research (JFCR), Koto-ku, Tokyo, 135-8550 Japan; 2 Graduate School of Life Science, Hokkaido University, Sapporo, Hokkaido 001-0021 Japan; 3 LSI Medience Corporation, Chiyoda-ku, Tokyo, 101-8517 Japan; 4 Department of Molecular Microbiology, Research Institute for Microbial Diseases (RIMD), Osaka University, Suita, Osaka 565-0871 Japan; 5 Division of Molecular Cell Engineering, Department of Genetics, National Institute of Genetics, ROIS, SOKENDAI, Mishima, Shizuoka 411-8540 Japan; 6 PRESTO, Japan Science and Technology Agency (JST), Kawaguchi, Saitama 332-0012 Japan; 7 CREST, Japan Agency for Medical Research and Development (AMED), Chiyoda-ku, Tokyo, 100-0004 Japan

Correction to: *Nature Communications*; 10.1038/ncomms15287; published online 16 May 2017.

This Article contains errors in Fig. 4. In panel d, the lanes of the western blot should have been labeled ‘1.05’,‘1.06, ‘1.09’, ‘1.11’ ‘1.13’, ‘1.16’, ‘1.19’, ‘1.22’, ‘1.24’, ‘1.25’. The correct version of Fig. 4 appears below.

**Fig. 4:**
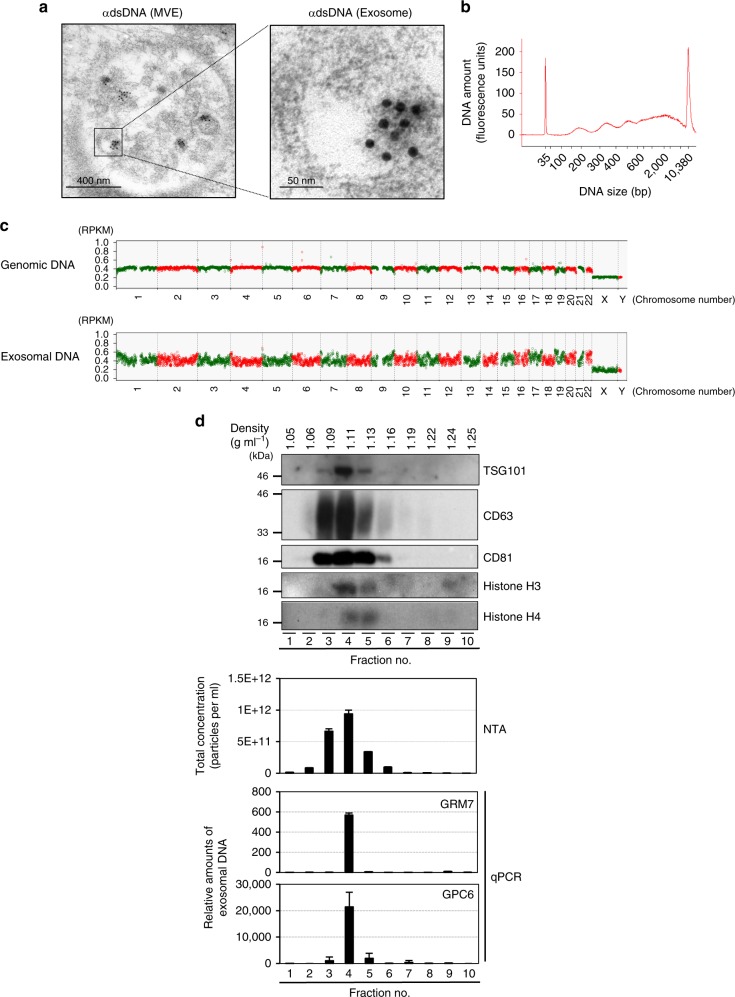
▓

