# Supplementary material for: Exosomes maintain cellular homeostasis by excreting harmful DNA from cells
Source: Nat Commun. 2017 May 16;8:15287. doi: 10.1038/ncomms15287 (PMC5440838; doi:10.1038/ncomms15287)
Supplement: Supplementary Information — Supplementary Figures, Supplementary Table and Supplementary References [file ncomms15287-s1.pdf]

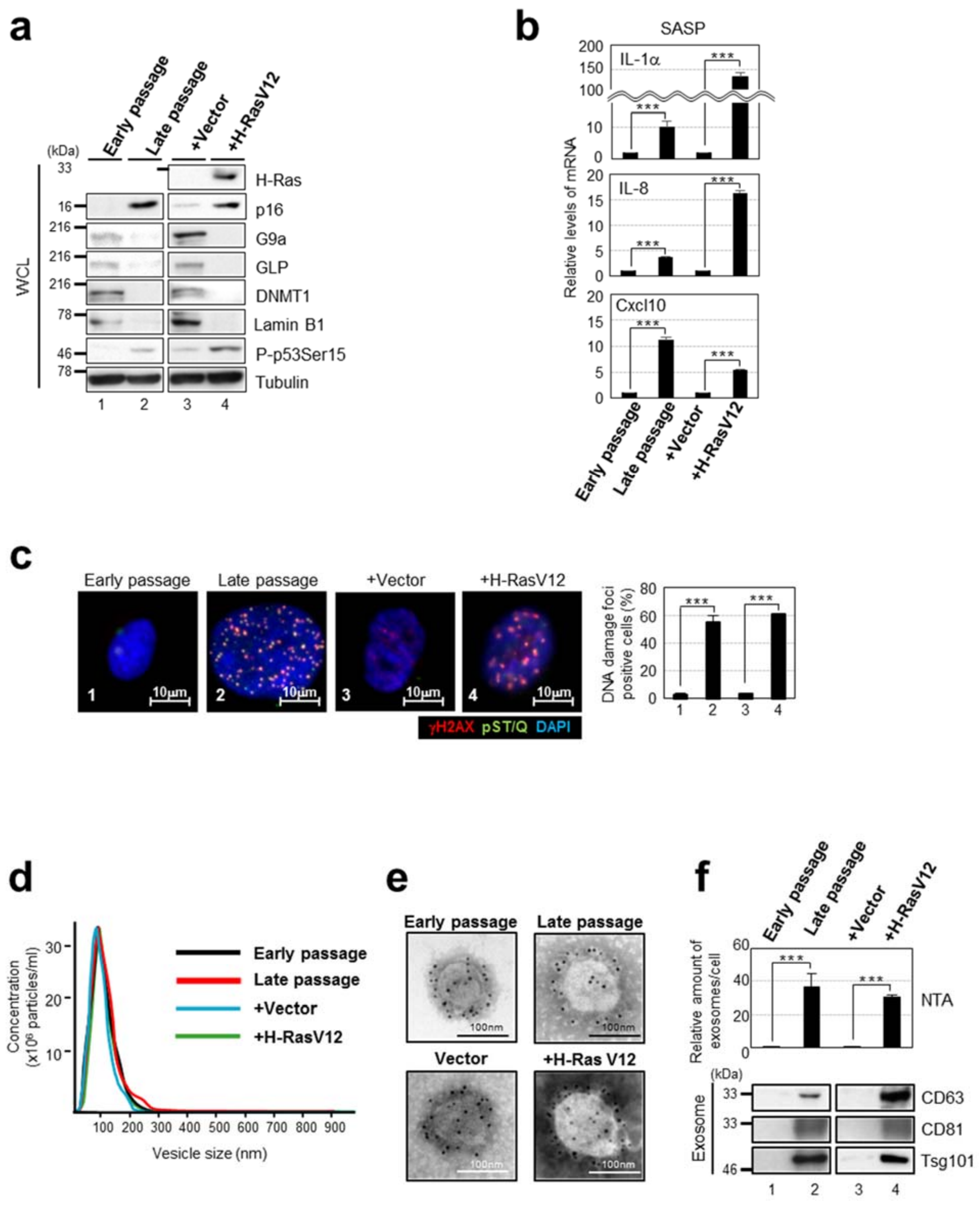

Supplementary Figure 1 | Induction of cellular senescence and isolation of exosome.
a to c, Pre-senescent primary normal human diploid fibroblasts (TIG-3 cells) were rendered senescent by either serial passage (lane 2) or ectopic expression of oncogenic

ras (lane 4), the most established ways to induce cellular senescence in culture[1-6]. These cells were then subjected to western blotting using antibodies shown right (**a**), qPCR analysis of SASP factor expression (**b**) or to immunofluorescence staining for markers of DNA damage (γ-H2AX [red], phosphor- Ser/Thr ATM/ATR (pST/Q) substrate [green] and DAPI [blue]) (**c**). The histograms indicate the percentage of nuclei that contain more than 3 foci positive for both γ-H2AX and pST/Q staining (**c**). At least 100 cells were scored per group (**c**). Tubulin was used as a loading control (**a**). The representative data from three independent experiments are shown. **d to f**, Isolated EVs from each cells population were confirmed to be exosomes based on the nanoparticle tracking (NanoSight) analysis (NTA) (**d**) and immuno-gold labelling for CD63, a well-known exosome-associated protein, followed by transmission electron microscopy (TEM) (**e**). Note that the same number of EVs from each samples were subjected to NTA (**d**). Relative amount of exosomes isolated from each cells population were examined by NTA and western blotting using antibodies against canonical exosome markers shown right (**f**). The representative data from three independent experiments were shown. For all graphs, error bars indicate mean ±standard deviation (s.d.) of triplicate measurements. (***$P<0.001$; one-way ANOVA). (WCL: whole cell lysates).

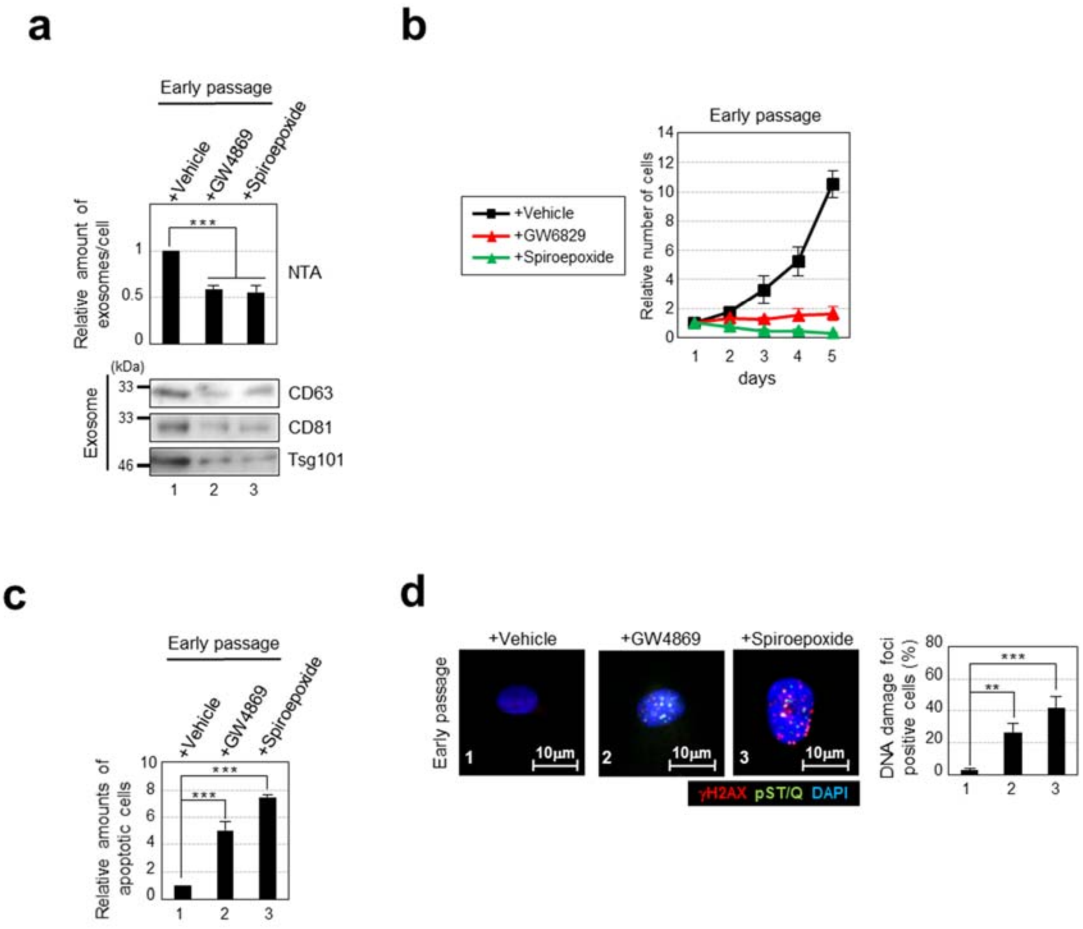

Supplementary Figure 2 | Inhibition of exosome secretion by chemical inhibitors in pre-senescent HDFs. Pre-senescent TIG-3 cells were treated for 48 h with control vehicle (DMSO) or with indicated chemical inhibitors (10 μM GW4869; 10 μM Spiroepoxide). These cells were then subjected to NanoSight (NTA) analysis and western blotting using antibodies against canonical exosome markers shown right for quantitative measurement of isolated exosome particles (**a**), cell proliferation analysis (**b**), apoptosis analysis at day 2 (**c**), or to immunofluorescence staining for markers of DNA damage (γ-H2AX [red], pST/Q [green] and DAPI [blue]) (**d**). The histograms indicate the percentage of nuclei that contain more than 3 foci positive for both γ-H2AX and pST/Q staining (**d**). At least 100 cells were scored per group (**d**). The representative data from three independent experiments are shown. For all graphs, error bars indicate mean ±standard deviation (s.d.) of triplicate measurements. (**P<0.01. ***P<0.001; one-way ANOVA).

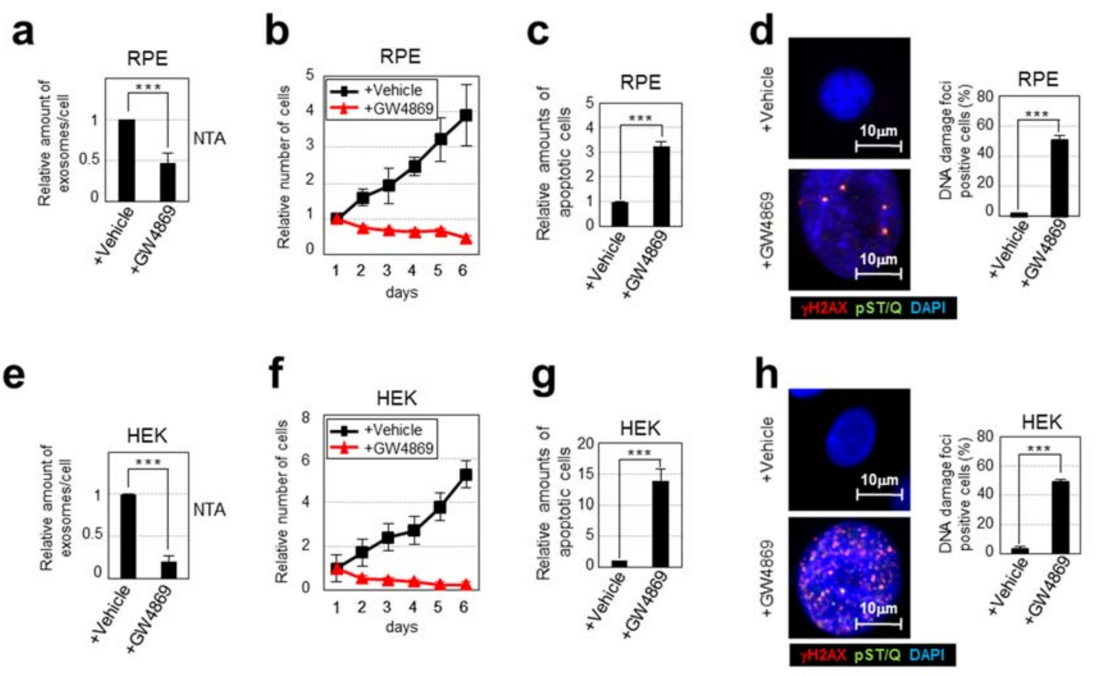

Supplementary Figure 3 | Inhibition of exosome secretion in primary normal human epithelial cells. Pre-senescent primary normal human retinal pigment epithelial (HRPE) cells (**a to d**) or pre-senescent normal human epidermal keratinocytes (HEK) (**e to h**) were treated for 48 h with control vehicle (DMSO) or with 5μM (HEK) or 10 μM (HRPE) of GW4869. These cells were then subjected to NanoSight analysis for quantitative measurement of isolated exosome particles (**a and e**), cell proliferation analysis (**b and f**), apoptosis analysis at day 2 (**c and g**) or to immunofluorescence staining for markers of DNA damage (γ-H2AX [red], pST/Q [green] and DAPI [blue]) (**d and h**). The histograms indicate the percentage of nuclei that contain more than 3 foci positive for both γ-H2AX and pST/Q staining (**d and h**). At least 100 cells were scored per group (**d and f**). The representative data from three independent experiments are shown. For all graphs, error bars indicate mean ±standard deviation (s.d.) of triplicate measurements. (***$P<0.001$; one-way ANOVA).

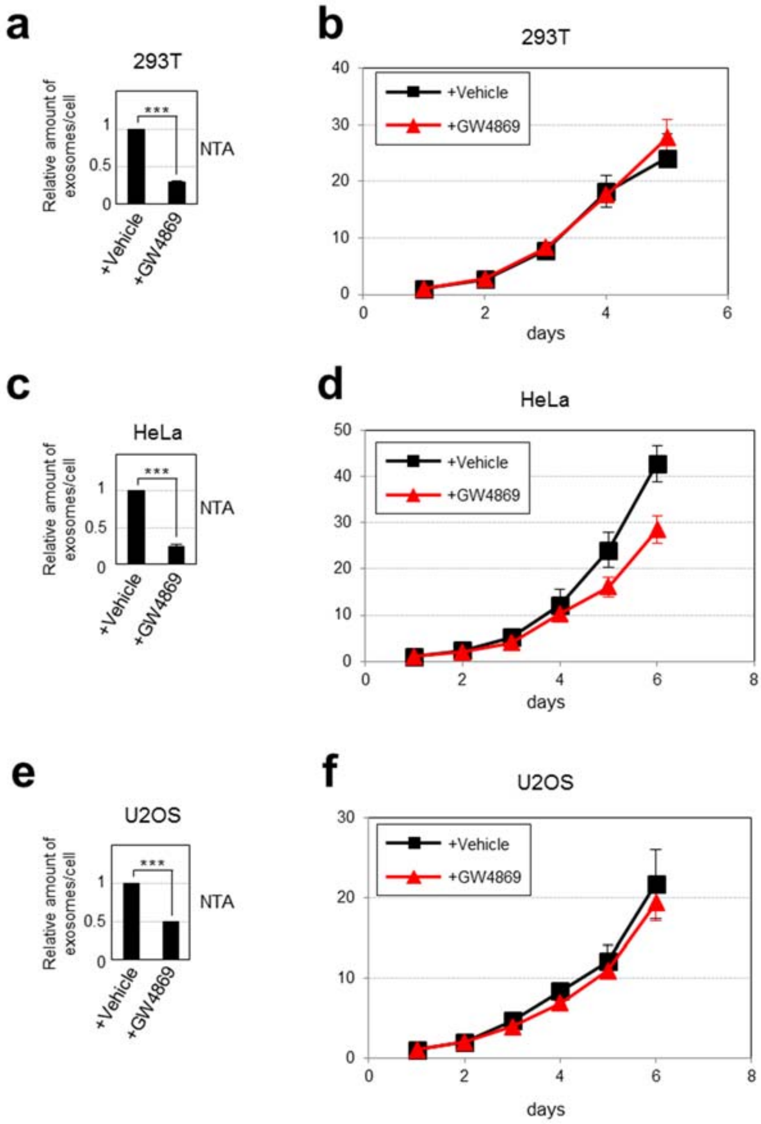

Supplementary Figure 4 | Inhibition of exosome secretion in human cancer cell lines. Human cancer cell lines indicated at the top of the panel were treated for 48 h with control vehicle (DMSO) or with 5 μM (for 293T cells) or 10 μM (for HeLa cell and U2OS cells) of GW4869. These cells were then subjected to NanoSight analysis for quantitative measurement of isolated exosome particles (**a, c and e**), or to cell proliferation analysis (**b, d and f**). The representative data from three independent experiments are shown. For all graphs, error bars indicate mean ±standard deviation (s.d.) of triplicate measurements. (***P<0.001; one-way ANOVA).

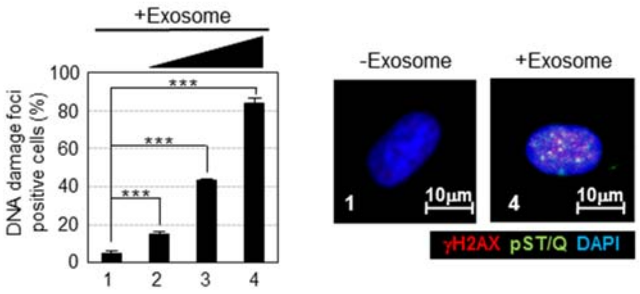

Supplementary Figure 5| Exosome has the potential to provoke the DDR in recipient cells. Pre-senescent TIG-3 cells were treated with increasing amounts of exosomes released from pre-senescent TIG-3 cells for 24h, and then subjected to immunofluorescence staining for markers of DNA damage (γ-H2AX [red], pST/Q [green] and DAPI [blue]). The representative data from three independent experiments are shown. The histograms indicate the percentage of nuclei that contain more than 3 foci positive for both γ-H2AX and pST/Q staining. Exosome concentrations used are as follows: Lanes 1 (0/ml), 2 (1.125 x 10^7/ml), 3 (2.25 x 10^7/ml), 4 (4.5 x 10^7/ml). At least 100 cells were scored per group. For all graphs, error bars indicate mean ±standard deviation (s.d.) of triplicate measurements. (***$P<0.001$; one-way ANOVA).

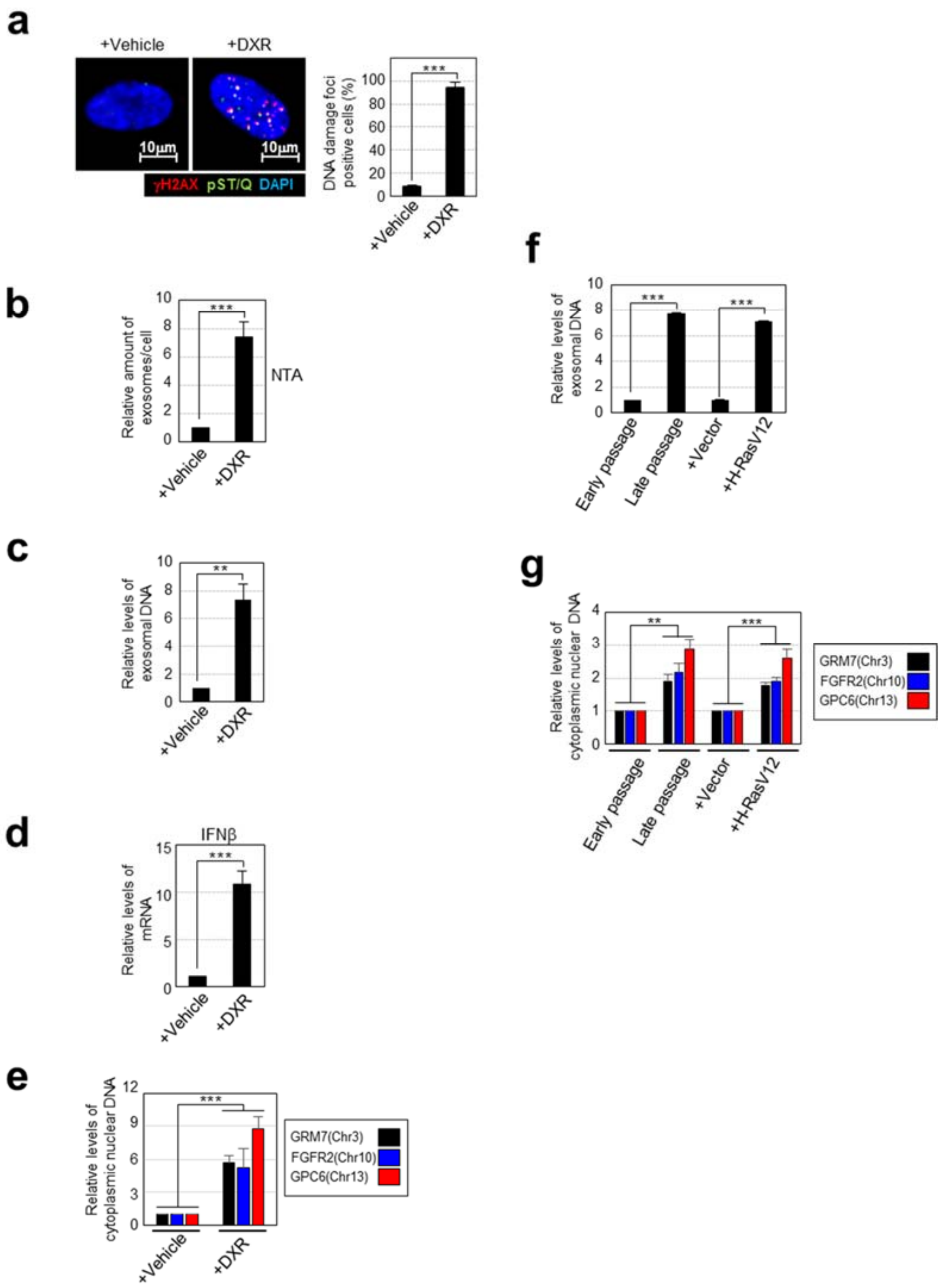

Supplementary Figure 6| DNA damage provokes cytoplasmic accumulation of nuclear DNA in HDFs. **a to e,** Pre-senescent TIG-3 cells were treated with control vehicle or 0.25 μg/ml of doxorubicin (DXR) for 10 days. These cells were then subjected to immunofluorescence staining for markers of DNA damage (γ-H2AX [red], pST/Q [green] and DAPI [blue]) (**a**), NanoSight analysis for quantitative measurement of isolated exosome particles (**b**), quantitative measurement of isolated exosomal DNA

using QuantiFluor dsDNA staining system (**c**), qPCR analysis of IFNβ gene expression (**d**) or to qPCR analysis of chromosomal DNA in cytoplasmic fraction (**e**). The histograms indicate the percentage of nuclei that contain more than 3 foci positive for both γ-H2AX and pST/Q staining (**a**). At least 100 cells were scored per group (**a**). The representative data from three independent experiments are shown. **f and g,** Pre-senescent TIG-3 cells were rendered senescent by either serial passage (late passage) or ectopic expression of oncogenic *ras* (+H-RasV12). These cells were then subjected to quantitative measurement of isolated exosomal DNA using QuantiFluor dsDNA staining (**f**), or to qPCR analysis of chromosomal DNA in cytoplasmic fraction (**g**). The representative data from three independent experiments are shown. For all graphs, error bars indicate mean ±standard deviation (s.d.) of triplicate measurements. (**P<0.01. ***P<0.001; one-way ANOVA).

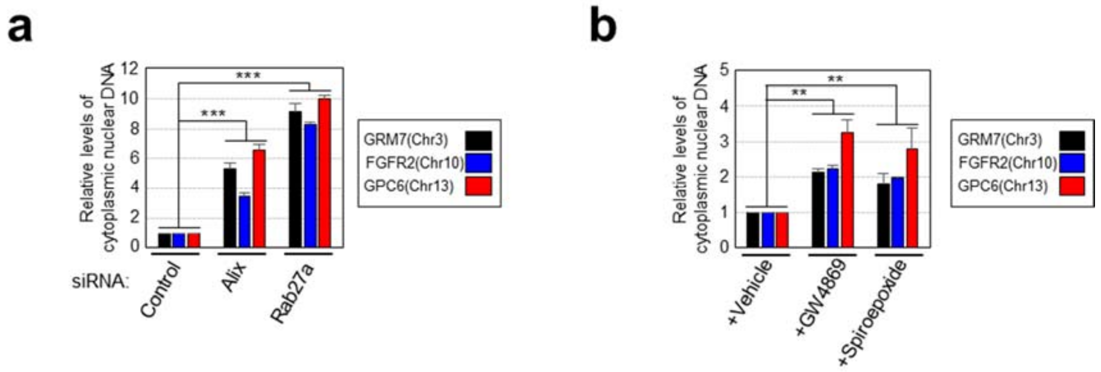

Supplementary Figure 7 | Inhibition of exosome secretion provokes cytoplasmic accumulation of nuclear DNA in HDFs. Exosome secretion was inhibited using indicated siRNA oligos (**a**) or chemicals (**b**) in pre-senescent TIG-3 cells as described in Fig.2a and Supplementary Fig. 2. These cells were then subjected to isolation of cytoplasmic fraction followed by qPCR analysis of chromosomal DNA. The representative data from three independent experiments are shown. For all graphs, error bars indicate mean ±standard deviation (s.d.) of triplicate measurements. (**P<0.01. ***P<0.001; one-way ANOVA).

a

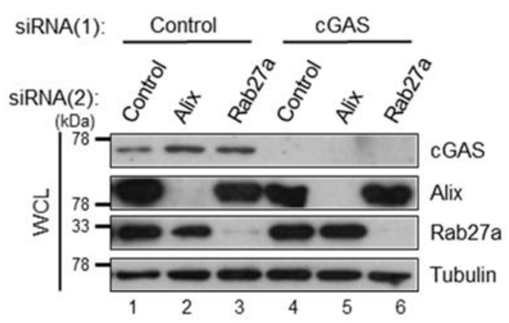

b

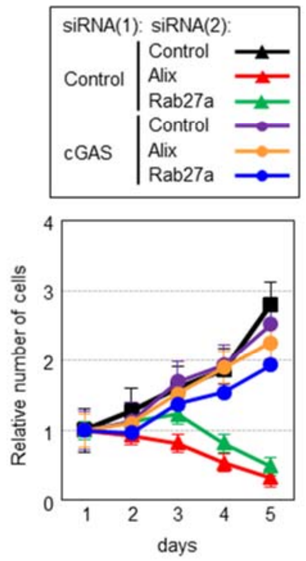

Supplementary Figure 8 | Depletion of cGAS attenuated the effects of Alix or Rab27a knockdown in HDFs. Pre-senescent TIG-3 cells were transfected with two different sets of validated siRNA oligos indicated at the top of the panel for three times at 2 day intervals. These cells were then subjected to western blotting using antibodies shown right (**a**), cell proliferation analysis (**b**). The representative data from three independent experiments are shown. For graph, error bars indicate mean $\pm$standard deviation (s.d.) of triplicate measurements. (WCL: whole cell lysates).

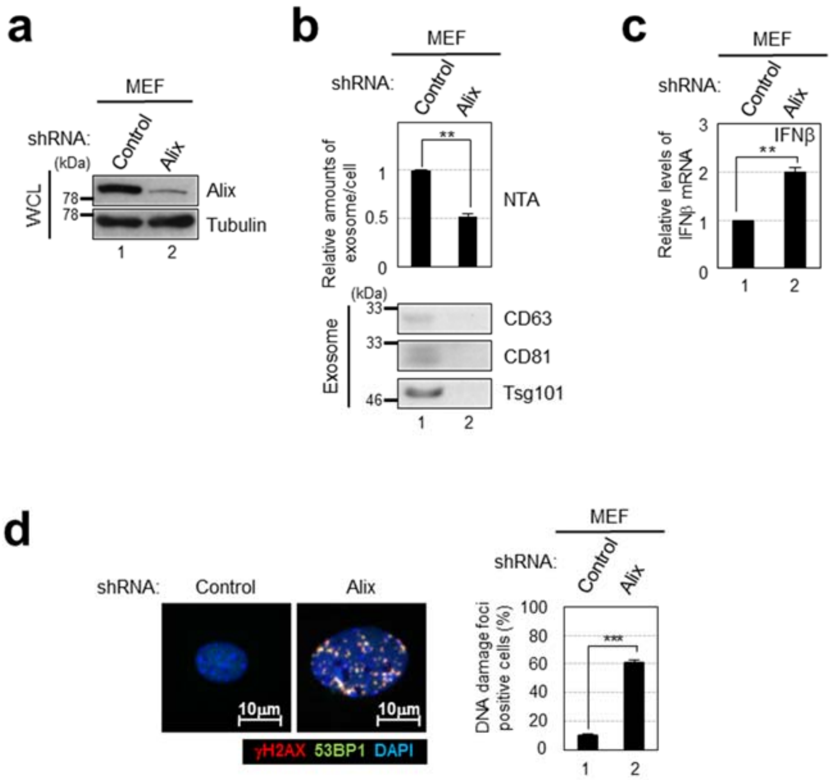

Supplementary Figure 9 | Inhibition of exosome secretion in primary MEFs. Early passage primary mouse embryonic fibroblasts (MEFs) were infected with retrovirus encoding shRNA against Alix or control three times. After puromycin selection, these cells were then subjected to western blotting using antibodies shown right (**a**), NanoSight analysis for quantitative measurement of isolated exosome particles and western blotting using antibodies against canonical exosome markers shown right (Exosome) (**b**), qPCR analysis of IFNβ gene expression (**c**) or to immunofluorescence staining for markers of DNA damage (γ-H2AX [red], 53BP1 [green] and DAPI [blue]) (**d**). The representative data from three independent experiments are shown. The histograms indicate the percentage of nuclei that contain more than 3 foci positive for both γ-H2AX and pST/Q staining. At least 100 cells were scored per group. For all graphs, error bars indicate mean ±standard deviation (s.d.) of triplicate measurements. (**P<0.01. ***P<0.001; one-way ANOVA).

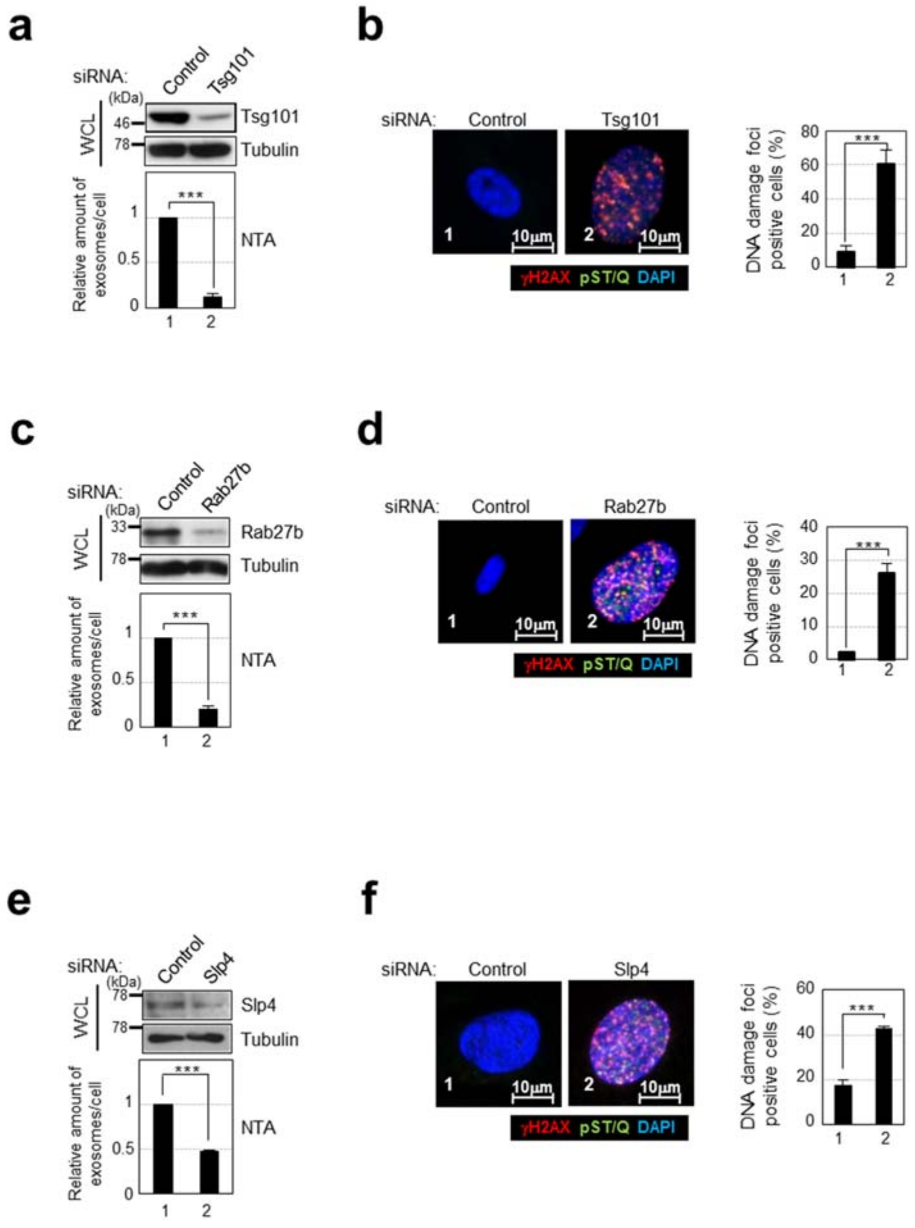

Supplementary Figure 10 | Inhibition of exosome secretion by knocking down Tsg101, Rab27b or Slp4 in HDFs. Pre-senescent TIG-3 cells were subjected to transfection with indicated siRNA oligos twice (at 2day intervals). These cells were then subjected to western blotting using antibodies shown right or to exosome isolation followed by NanoSight analysis (NTA) for quantitative measurement of isolated exosome particles (**a, c, e**), followed by immunofluorescence staining for markers of DNA damage (γ-H2AX [red], phosphor-Ser/Thr ATM/ATR (pST/Q) substrate [green] and DAPI [blue]) (**b, d, f**). Tubulin was used as a loading control. The representative data from three independent experiments are shown. The histograms indicate the

percentage of nuclei that contain more than 3 foci positive for both γ-H2AX and pST/Q staining (**b, d, f**). At least 100 cells were scored per group (**b, d, f**). (WCL: whole cell lysates).

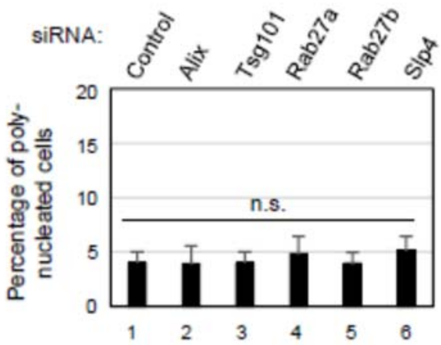

Supplementary Figure 11 | Depletion of Alix does not cause cytokinetic failure in HDFs. Pre-senescent TIG-3 cells were subjected to transfection with indicated siRNA oligos twice (at 2day intervals). These cells were then subjected to DAPI staining and multinucleated cells were counted. The representative data from three independent experiments are shown. The histograms indicate the percentage of cells that contain more than 2 nuclei. At least 100 cells were scored per group. For all graphs, error bars indicate mean ±standard deviation (s.d.) of triplicate measurements. (n.s. = not significant; one-way ANOVA)

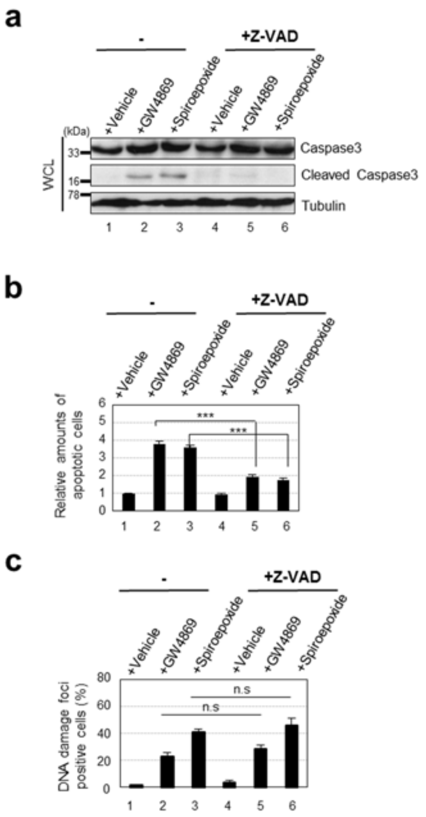

Supplementary Figure 12 | Inhibition of apoptosis does not have any impact on the appearance of DDR in cells with exosome inhibitors. Pre-senescent TIG-3 cells were treated for 48 h with control vehicle (DMSO), GW4869 (10μM) or Spiroepoxide (10μM) in the presence or absence of 20μM Z-VAD. These cells were then subjected to western blotting using antibodies shown right (**a**), apoptosis analysis (**b**), immunofluorescence staining for markers of DNA damage (γ-H2AX [red], pST/Q [green] and DAPI [blue]) (**c**). The histograms indicate the percentage of nuclei that contain more than 3 foci positive for both γ-H2AX and pST/Q staining (**c**). At least 100 cells were scored per group (**c**). The representative data from three independent experiments are shown. For all graphs, error bars indicate mean ±standard deviation (s.d.) of triplicate measurements. (**P<0.01. ***P<0.001. n.s. = not significant; one-way ANOVA). (WCL: whole cell lysates).

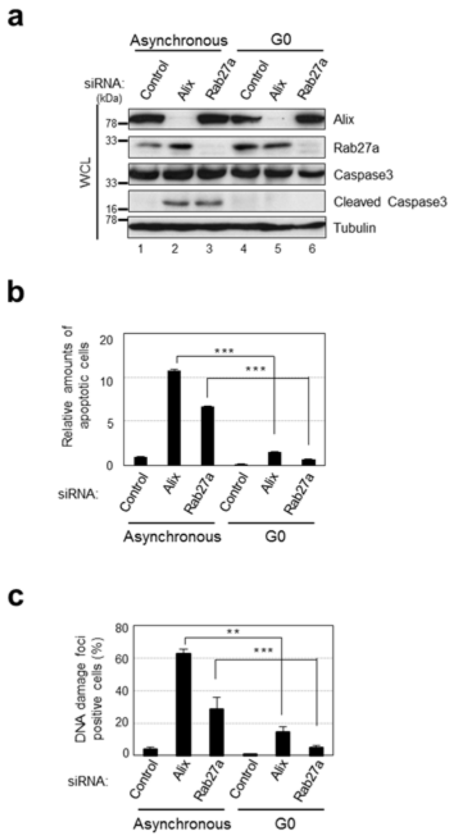

Supplementary Figure 13 | Cells in G0 phase are more resistant to inhibition of exosome secretion in HDFs. Asynchronously proliferating TIG-3 cells or TIG-3 cells rendered quiescent (G0 phase) by contact inhibition were transfected with validated siRNA oligos indicated at the top of the panel for two times at 2 day intervals. These cells were then subjected to western blotting using antibodies shown right (**a**), apoptosis analysis (**b**), immunofluorescence staining for markers of DNA damage (γ-H2AX [red], pST/Q [green] and DAPI [blue]) (**c**). The histograms indicate the percentage of nuclei that contain more than 3 foci positive for both γ-H2AX and pST/Q staining (**c**). At least 100 cells were scored per group (**c**). The representative data from three independent experiments are shown. For all graphs, error bars indicate mean ±standard deviation (s.d.) of triplicate measurements. (***P<0.001; one-way ANOVA). (WCL: whole cell lysates).

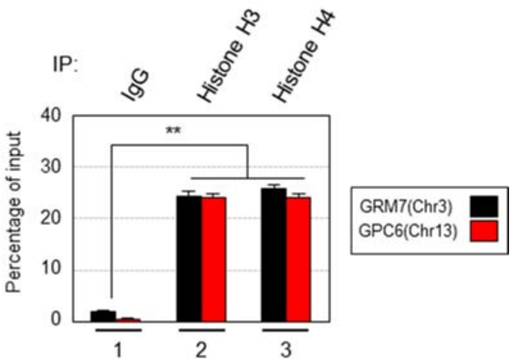

Supplementary Figure 14 | Exosomal DNAs were bound to histones. Exosome components prepared from pre-senescent TIG-3 cells were subjected to DNA-protein cross-linking followed by immuno-precipitation using antibodies against Histone H3, Histone H4 or control IgG. The presence of indicated genomic DNA fragments in the immuno-precipitated complexes were measured by qPCR. The representative data from three independent experiments are shown. Error bars indicate mean ±standard deviation (s.d.) of triplicate measurements. (**P<0.01; one-way ANOVA)

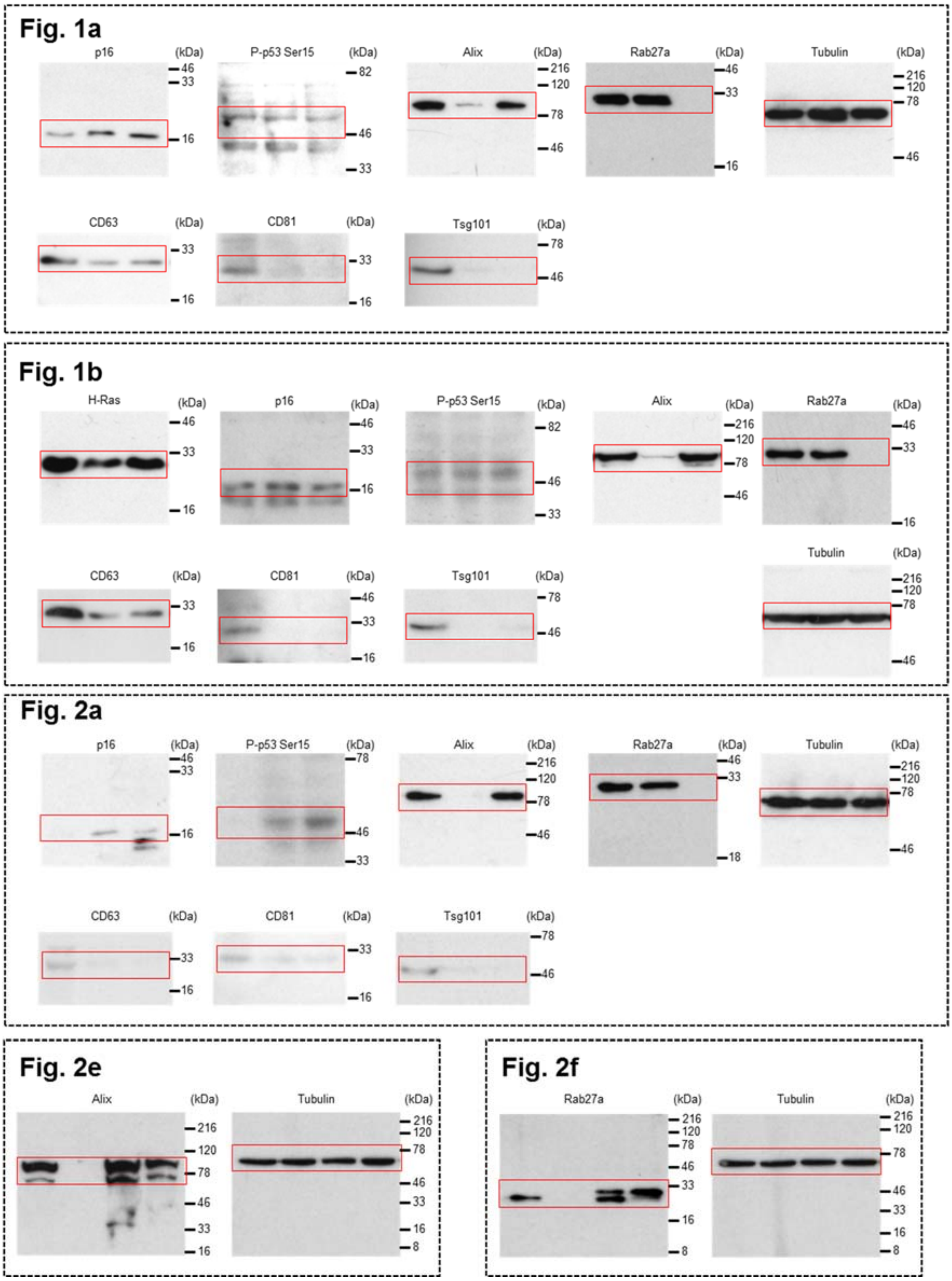

Supplementary Figure 15 | The original immunoblot images.

Fig. 3a

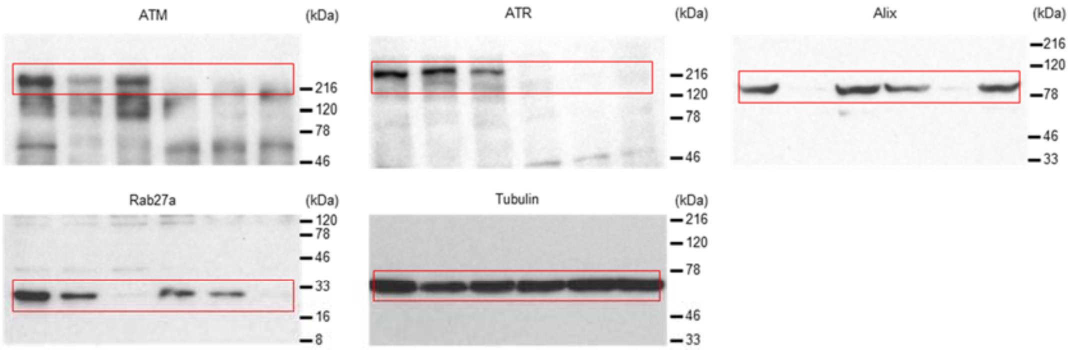

Fig. 4d

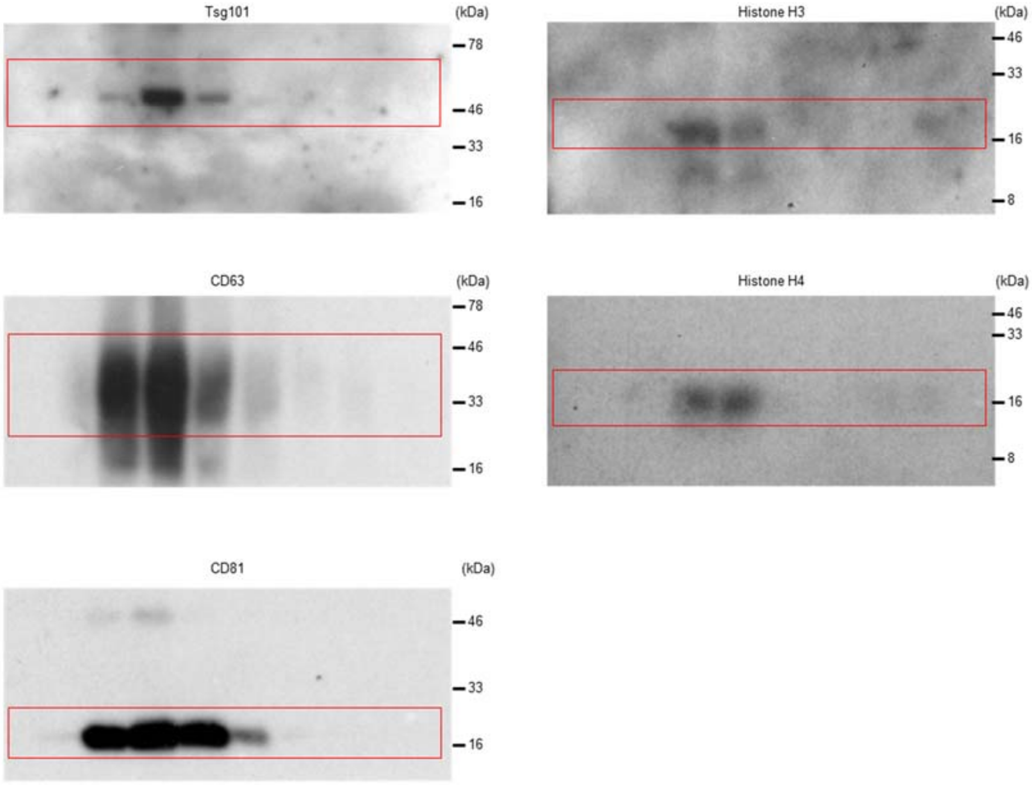

Supplementary Figure 15 | continued.

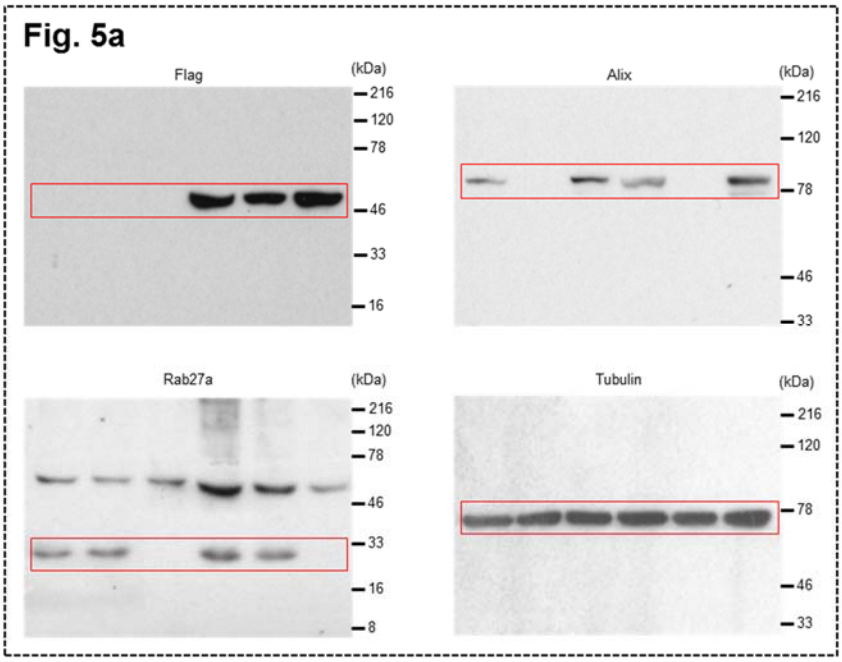

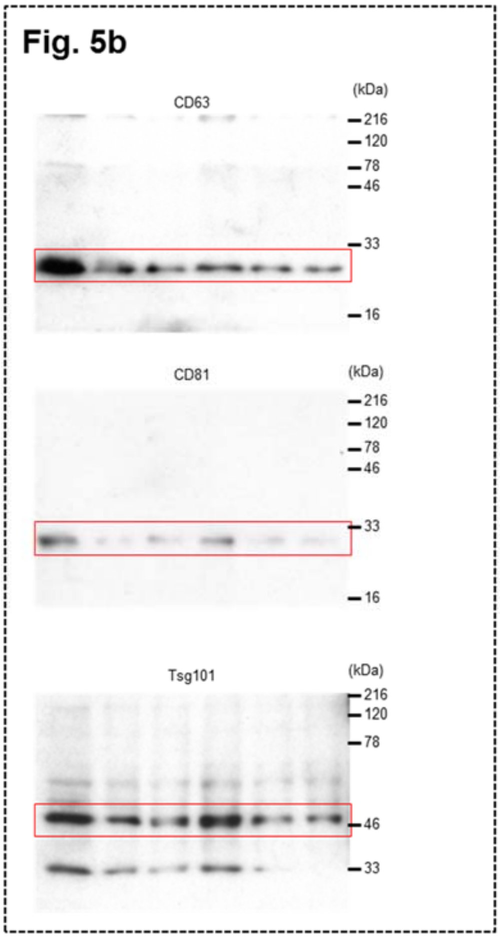

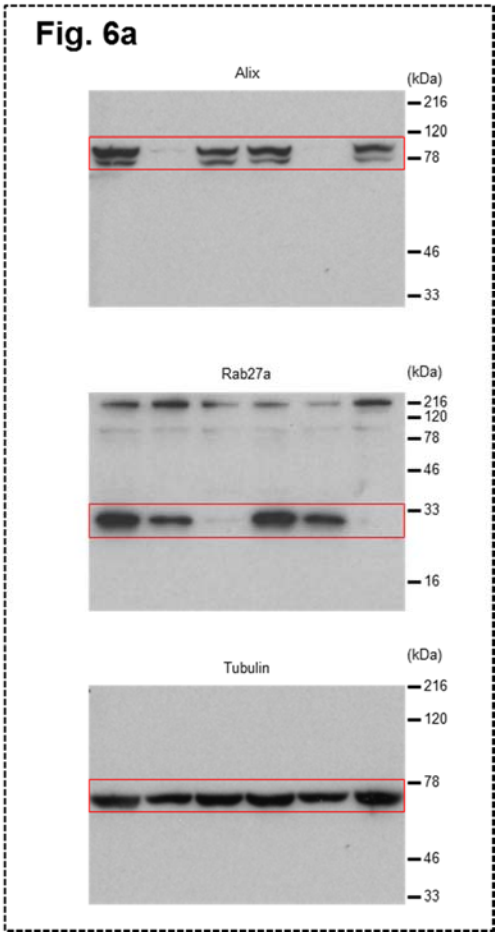

Supplementary Figure 15 | continued.

Fig. 7a

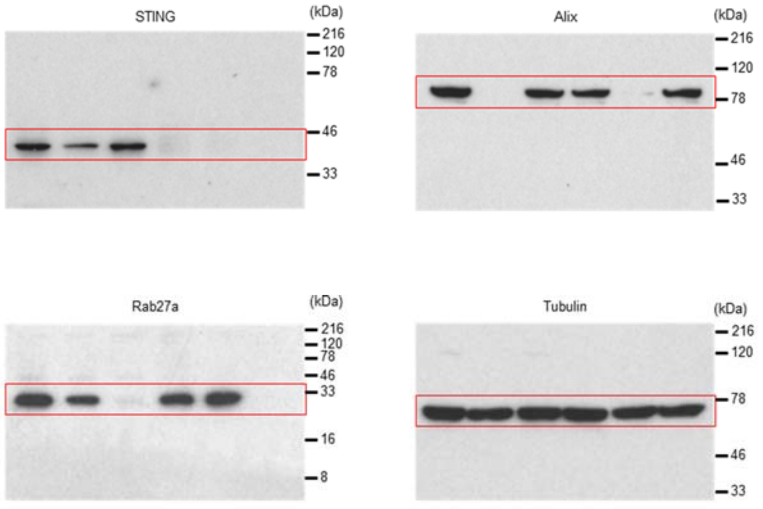

Fig. 8b

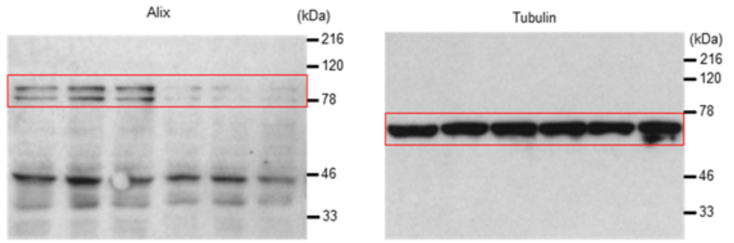

Fig. 9b

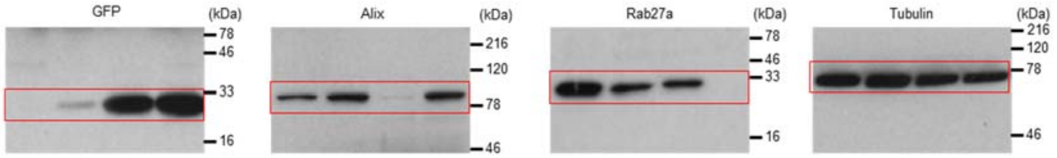

Supplementary Figure 15 | continued.

Fig. 9c

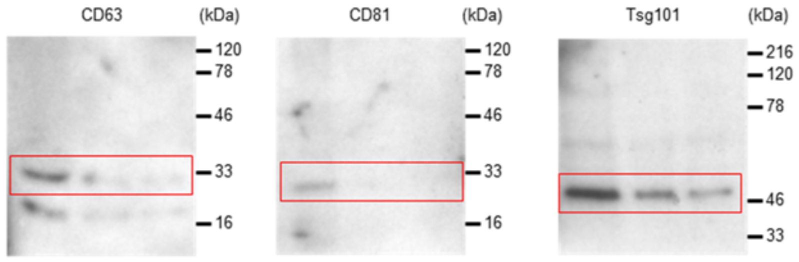

Fig. 9g

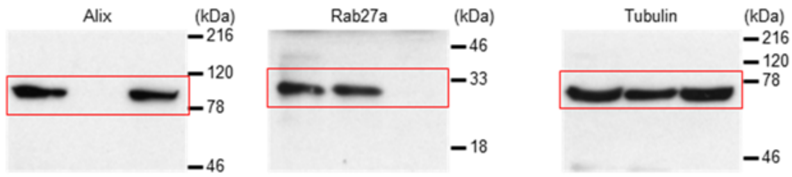

Fig. 9h

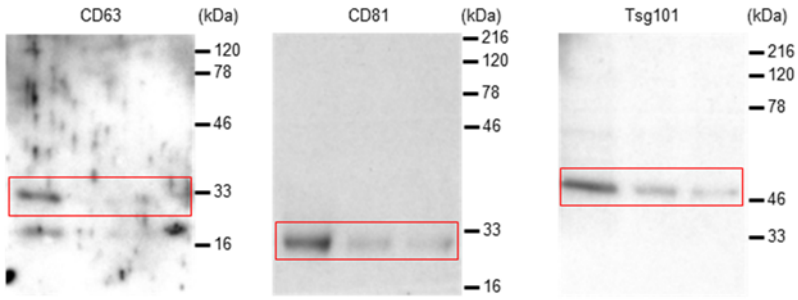

Supplementary Figure 15 | continued.

Quantitative Real-Time PCR primers			
**Gene**		**Primer**	**Reference No.**
human GAPDH	*F*	5’-CAACTACATGGTTTACATGTTC-3’	5, 42
	R	5’-GCCAGTGGACTCCACGAC-3’	
human IL-1α	*F*	5’-AACCAGTGCTGCTGAAGGA-3’	42
	R	5’-TTCTTAGTGCCGTGAGTTTCC-3’	
human IL-8	*F*	5’-AAGGAAAACTGGGTGCAGAG-3’	42
	R	5’-ATTGCATCTGGCAACCCTAC-3’	
human CXCL10	*F*	5’-CCAGAATCGAAGGCCATCAA-3’	1*
	R	5’-CATTTCCTTGCTAACTGCTTTCAG-3’	
human IFN-β	*F*	5’-AAACTCATGAGCAGTCTGCA-3’	66
	R	5’-AGGAGATCTTCAGTTTCGGAGG-3’	
human GRM7	*F*	5’-TCAAGTGCCACATCCTATGC-3’	
	R	5’-ATTTTTCTAGCCAGGCACCA-3’	
human FGFR2	*F*	5’-ACCTGGAAATGGCTGAAATG-3’	
	R	5’-AAGTCCTCGCAGAGGTTTCA-3’	
human GPC6	*F*	5’-CGCCAGTGTGTGTAGCACTT-3’	
	R	5’-TCGGCCTCTCTCAGTTCTGT-3’	
Adenovirus	*F*	5’-TTCCCCATGGCTCACAACAC-3’	
	R	5’-TGACGGCTCATGGGCTGGAAGTT-3’	
GFP	*F*	5’-CAACAGCCACAACGTCTATATCATG-3’	2*
	R	5’-ATGTTGTGGCGGATCTTGAAG-3’	
mouse GAPDH	*F*	5’- CAACTACATGGTCTACATGTTC-3’	14, 42
	R	5’- CGCCAGTAGACTCCACGAC-3’	
mouse IFN-β	*F*	5’- CAGCTCCAAGAAAGGACGAAC-3’	3*
	R	5’- GGCAGTGTAACTCTTCTGCAT-3’	

*Supplementary references

Supplementary Table 1 | List of Quantitative Real-Time PCR primers

Supplementary References

1 Khan, S. *et al.* Differential gene expression of chemokines in KRAS and BRAF mutated colorectal cell lines: role of cytokines. *World J. Gastroenterol.* **20**, 2979-2994 (2014).

2 Ghosh, M., Liu, G., Randall, G., Bevington, J. & Leffak, M. Transcription factor binding and induced transcription alter chromosomal c-myc replicator activity. *Mol. Cell. Biol.* **24**, 10193-10207.2004 (2004).

3 Turer, E. E. *et al.* Homeostatic MyD88-dependent signals cause lethal inflamMation in the absence of A20. *J. Exp. Med.* **205**, 451-464 (2008).
